# A nearly complete haplotype-phased genome assembly of nerve plant (*Fittonia albivenis*) provides insights into leaf color evolution

**DOI:** 10.1093/hr/uhaf154

**Published:** 2025-06-26

**Authors:** Longxin Wang, Kai-Hua Jia, Ren-Gang Zhang, Chenyang Hao, Xiaochun Qin

**Affiliations:** School of Biological Science and Technology, University of Jinan, Jinan, China; Institute of Crop Germplasm Resources, Shandong Academy of Agricultural Sciences, Jinan, China; State Key Laboratory of Plant Diversity and Specialty Crops/Yunnan Key Laboratory for Integrative Conservation of Plant Species with Extremely Small Populations, Kunming Institute of Botany, Chinese Academy of Sciences, Kunming, China; School of Biological Science and Technology, University of Jinan, Jinan, China; School of Biological Science and Technology, University of Jinan, Jinan, China

## Abstract

*Fittonia albivenis*, commonly known as the nerve plant, is an ornamental species native to the Peruvian rainforest, valued for its vibrant and diverse leaf coloration. Understanding the genetic mechanisms underlying this coloration is crucial for enhancing its ornamental value and adaptation to environmental stressors. Here, by leveraging advanced sequencing technologies such as PacBio HiFi, Oxford Nanopore, and Hi-C, we achieved a nearly complete haplotype-phased genome assembly for *F. albivenis*, revealing a 2.08-Gb genome composed of 18 chromosome pairs and containing 66 telomeres. This assembly enabled the identification of subgenome-specific repetitive sequences, elucidating their impact on gene expression and structural variations. Through RNA sequencing, metabolomic profiling, and resequencing, we dissected the regulatory networks influencing chlorophyll and anthocyanin biosynthesis, identifying key genes and transcription factors driving leaf color variation. Our findings highlight the roles of gene duplication and specific transcription factors in pigment synthesis pathways, providing a foundation for future genetic studies and breeding programs aimed at enhancing ornamental and adaptive traits in *F. albivenis* and related species.

## Introduction

Leaves, as a vital component of photosynthesis, are essential for plant growth and development. Leaf color mutations, a common and easily identifiable trait variation across many higher plants, have a pronounced effect on photosynthetic efficiency due to their impact on photosynthetic pigments, which are critical for light energy conversion. These pigments, including chlorophyll and carotenoids, play key roles in capturing and utilizing light energy. Mutations in leaf color often alter the synthesis, degradation, content, and ratio of plant pigments, including photosynthetic pigments, anthocyanins, flavonols, and xanthophylls, which disrupt the photosynthetic process and alter the ability to cope with photooxidative damage. This disturbance frequently leads to impaired growth or even plant death, as leaf color mutations hinder the plant’s ability to effectively convert light into chemical energy. Despite their historically perceived lack of practical value [1], leaf color mutants are now recognized as valuable research models for investigating the mechanisms of photosynthesis, the biosynthetic pathways of pigments, chloroplast development, and the genetic regulation of these processes [2,3].

*Fittonia albivenis*, commonly known as the nerve plant, thrives as an understory perennial in the dense Peruvian rainforest. Its remarkably colorful foliage, which includes an array of hues from deep greens accented with white, red, pink, to bright red, yellow, and cream venation, primarily results from the presence of chlorophyll and anthocyanin. These pigments not only enhance the plant’s aesthetic appeal but also play vital roles in resistance to both biotic and abiotic stresses [4–6]. The complexity of leaf color variations in *F. albivenis* underscores its potential as a model organism for investigating the evolution of leaf coloration.

Decoding complete genomic sequences facilitates the detection of genetic variations within the genome, providing a foundational resource for exploring variations in leaf coloration. With advancements in single-molecule sequencing technologies such as PacBio High-Fidelity (HiFi) and ultra-long Oxford Nanopore Technologies (ONT), coupled with high-throughput chromatin conformation capture (Hi-C) sequencing and assembly techniques, an increasing number of telomere-to-telomere (T2T) genomes are being decoded, achieving the ultimate goal of genome assembly [6]. Typically, for highly heterozygous species, it is feasible to combine haplotype-resolved genome assembly with T2T assembly to construct a haplotype-resolved T2T genome. Alternatively, sequencing the F1 progeny from the hybridization of two closely related species with distinct life histories enables the simultaneous capture of both species’ genomes. Subsequently, by using Subphaser [7], which is based on species- or subgenome-specific sequences, the two genome sequences can be accurately phased, and the resulting genome assembly is referred to as a haplotype-phased genome. This approach provides comprehensive genetic information for both species, enhancing our understanding of genetic diversity and evolutionary dynamics between closely related species. High-quality haplotype-phased genome assemblies are crucial for resolving interspecies structural variations [8,9], telomere and centromere sequence variations [10,11], and allele-specific expression [12]. Together with transcriptomic and metabolomic technologies, these assemblies facilitate the identification of key genes, enhancing our understanding of the regulatory mechanisms behind secondary metabolite biosynthesis.

In this study, we focused on leveraging these advanced genomic and bioinformatic tools to deepen our understanding of the genetic architecture underlying chlorophyll and anthocyanin accumulation in *F. albivenis*. We assembled a nearly complete haplotype-phased genome, which facilitated precise genetic studies and the identification of variant genetic elements influencing phenotype. Notably, we identified subgenome-specific amplification of repetitive sequences that appear to affect the expression of adjacent genes. Through RNA sequencing (RNA-seq) and metabolomic profiling of differentially colored leaves, coupled with *k*-means analysis, we dissect the regulatory networks influencing chlorophyll and anthocyanin pathways. We observed a notable increase in the variation of key genes across the population. This comprehensive genomic exploration not only sheds light on the chlorophyll and anthocyanin biosynthesis in *F. albivenis* but also sets a foundation for future genetic studies and breeding programs aimed at enhancing the ornamental and adaptive traits of this and related species.

## Results

### A nearly complete and haplotype-phased genome assembly

To mitigate bias introduced by polymerase chain reaction (PCR) duplication in genome size estimation, we generated 103 Gb of PCR-free DNBSEQ data for our survey, revealing an approximate genome size of 1.2 Gb with a heterozygosity rate exceeding 1% (Supplementary Fig. S1). To achieve a more complete genome assembly for the F1 hybrid individual, we sequenced 64 Gb (~60× coverage) of PacBio HiFi long reads and 76 Gb (70× coverage) of ONT ultralong reads, along with 145 Gb of Hi-C data (Supplementary Table S1). Since the F1 hybrid individual and the survey plant are not identical, we conducted a resurvey using HiFi data from the F1 hybrid individual, which revealed a heterozygosity of 2.87% (Supplementary Fig. S2), Furthermore, based on HiFi data, we inferred that the genome sequencing sample is diploid using Smudgeplot [13] (Supplementary Fig. S3).

**Figure 1:**
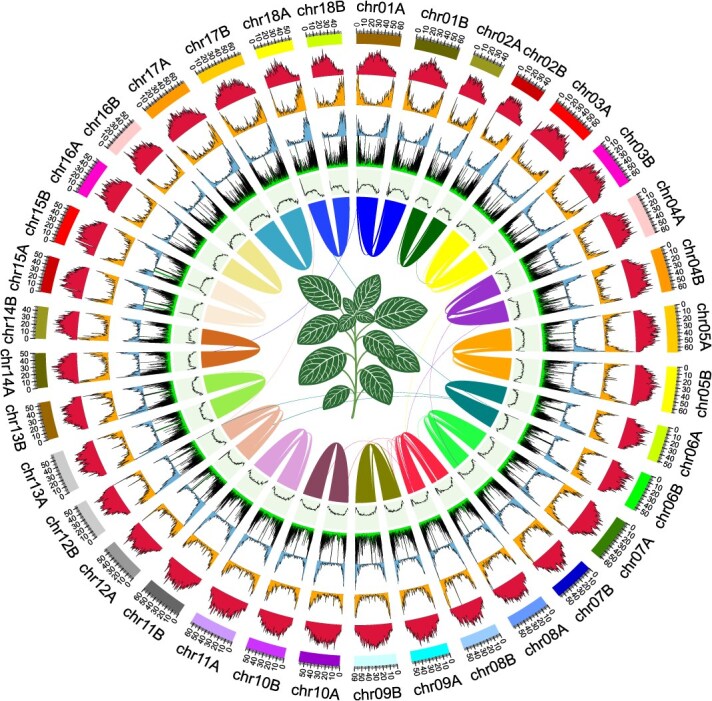
Synteny and distributions for genomic features of *F. albivenis*. Concentric circles—from outermost to innermost—Assembled chromosomes, Class I of TE density, Class II of TE density, Gene density, Tandem repeat density, GC content. Counts were calculated for every 100 kb. Lines in the center of the circle represent chromosome collinearities. The plant in the middle was used as a sample for the genome assembly.

Initially, HiFi reads were assembled into contigs using Hifiasm [14], followed by haplotype assembly for further analysis. After the initial assembly, only six gaps remained (Supplementary Table S2). Hi-C paired-end reads were then aligned to the haplotype assembly using Juicer [15], with manual inspection and adjustments made using Juicebox [16] to correct various errors, such as misinsertions, ultimately creating a chromosomal framework. Next, ONT ultralong reads were assembled into contigs (ONT-contigs) using NextDenovo [17], and these ONT-contigs were mapped to the previously assembled chromosomes using Unimap (https://github.com/lh3/unimap) to bridge gaps. At this point, the genome was assembled into a 0-gap structure comprising 18 pairs of chromosomes (Fig. 1). We manually checked these regions using the genome browser IGV and found that ONT-contigs can span these regions without any abnormalities (Supplementary Fig. S4).

To extend the chromosome ends and maximize chromosomal length, reads aligned to chromosome ends were reassembled using Hifiasm and mapped back to the chromosomes. Additionally, the mitochondrial and chloroplast genomes were assembled using GetOrganelle [18]. As a result, we achieved a nearly complete assembly for the *F. albivenis* genome, comprising 18 pairs of chromosomes and a total genome length of 2.08 Gb. The contig and scaffold N50 values reached 57.89 Mb, and a total of 66 telomeric sequences (TTTAGGG/CCCTAAA) were assembled (Table 1, Supplementary Fig. S5).

**Table 1:** Genome assembly statistics

Features	Statistics
Genome size (Gb)	2.08
Chromosome number	36
GC content (%)	38.72
Contig number (%)	45
Contig N50 (Mb)	57.89
Genome BUSCO (%)	99.2
Repeat (%)	72.83
LTR (%)	60.61
Gene number	56 094
annotated gene (%)	99.03
proteome BUSCO (%)	98.1

Subsequently, we utilized the *k*-mer-based method Subphaser [7] to phase two sets of subgenomes, identifying a significant amount of subgenome-specific sequence expansions that allowed phasing, leading to the subgenomes being designated as A and B (Supplementary Fig. S6). Accordingly, we refer to this genome assembly as ‘haplotype-phased’.

### Genome annotation

To identify transposable elements (TEs) *de novo*, we utilized EDTA [19] to create a TE library and employed RepeatMasker [20] for identifying repetitive regions in the genome. In total, we identified 2 119 690 repetitive sequences, spanning 1 517 989 759 bp, which constituted 72.8% of the total genome length (Table 1, Supplementary Table S3). The predominant category was long terminal repeat (LTR) elements, accounting for 60.6% of the repeats, with the majority being Gypsy elements. These Gypsy elements displayed a chromosomal distribution decreasing from the center toward the ends, comprising 44.9% of the genome. Additionally, 6.4% were Copia elements, with the remaining 9.3% being unidentified LTR elements. Beyond LTRs, we also discovered that DNA-type repetitive elements constituted 7.9% of the genome, predominantly located in specific genomic regions.

To better annotate functional genes, we generated 10 G of full-length transcriptomic data using the ONT platform (Supplementary Table S1). These reads were aligned to the genome using Minimap2 [21] and assembled using StringTie [22] as transcriptomic evidence. For gene annotation, we utilized 283 692 nonredundant protein sequences as homologous protein evidence. Ultimately, combining transcriptomic evidence, protein evidence, and *ab initio* predictions, we identified 56 094 protein-coding gene sequences, 9479 rRNA sequences, 3337 tRNA sequences, and 1171 noncoding RNA (ncRNA) sequences (Table 1, Supplementary Table S4).

**Figure 2:**
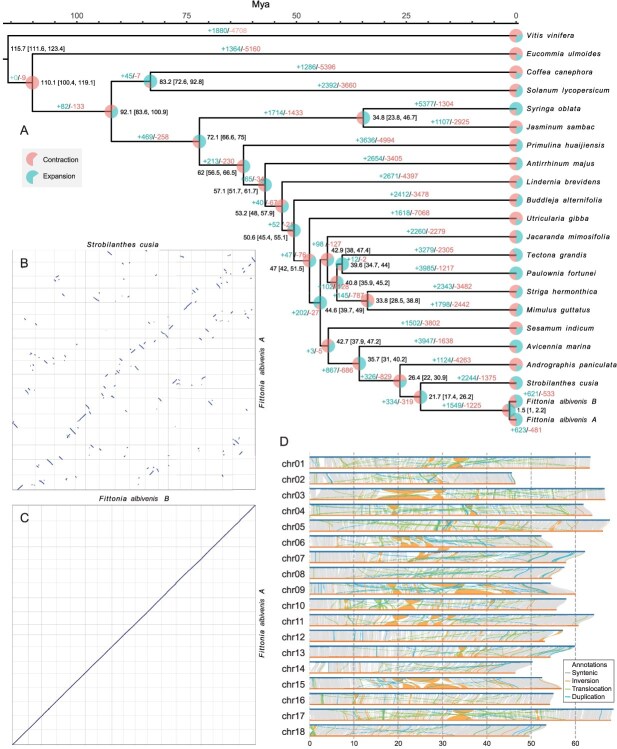
Comparative genomic analysis of *F. albivenis*. (A) Phylogenetic tree showing the divergence times and evolutionary relationships among *F. albivenis* and related species. The timeline at the top indicates millions of years ago (Mya). The circles at each node represent the contraction (red) and expansion (blue) of gene families. The numbers within the boxes indicate divergence time estimates with 95% confidence intervals in brackets, and the numbers in green and red represent expanded and contracted gene families, respectively. (B) Dot plot comparing the genomes of *S. cusia* and *F. albivenis* B, highlighting syntenic blocks and structural variations. (C) Dot plot comparing the subgenomes of *F. albivenis* A and *F. albivenis* B, showing a high degree of collinearity. (D) Synteny analysis between the chromosomes of *F. albivenis* A and *F. albivenis* B. Colored lines within the plot indicate different types of structural variations: syntenic regions (blue), inversions (orange), translocations (green), and duplications (purple).

We performed functional annotation of these protein-coding genes using three strategies: comparison with the eggNOG orthologous gene database, various protein databases, and subdatabases of InterPro. Our findings indicated that 99.03% of the genes were functionally annotated. Specifically, the Gene Ontology (GO) database annotated 48.85% of the protein-coding genes, the Kyoto Encyclopedia of Genes and Genomes (KEGG) database annotated 46.34%, and the Pfam database annotated 82.02% (Table 1, Supplementary Table S5).

### Assessment of the nearly complete genome completeness

We employed BUSCO [23], utilizing the embryophyta_odb10 lineage dataset, to assess the completeness of our genome and protein-coding genes. Of the 1614 core genes in this dataset, 99.2% were identified as complete within the genome, and 98.1% were found complete within the protein-coding genes (Table 1, Supplementary Table S6). Additionally, we utilized the *k*-mer-based Merqury software [24] to evaluate the quality of our genome assembly, achieving a high-quality score of 98.53 and a *k*-mer completeness assessment score of 55.01 (Table 1, Supplementary Fig. S7). We further aligned HiFi and ONT data back to the genome and observed that >97.5% of reads could be mapped back to the genome, with >99.7% of genomic positions having coverage of >10× reads (Supplementary Table S7). Lastly, the alignment of Hi-C data to the genome revealed no major visible errors (Supplementary Fig. S8). These results indicate that our genome assembly and annotation are both highly complete and accurate.

**Figure 3:**
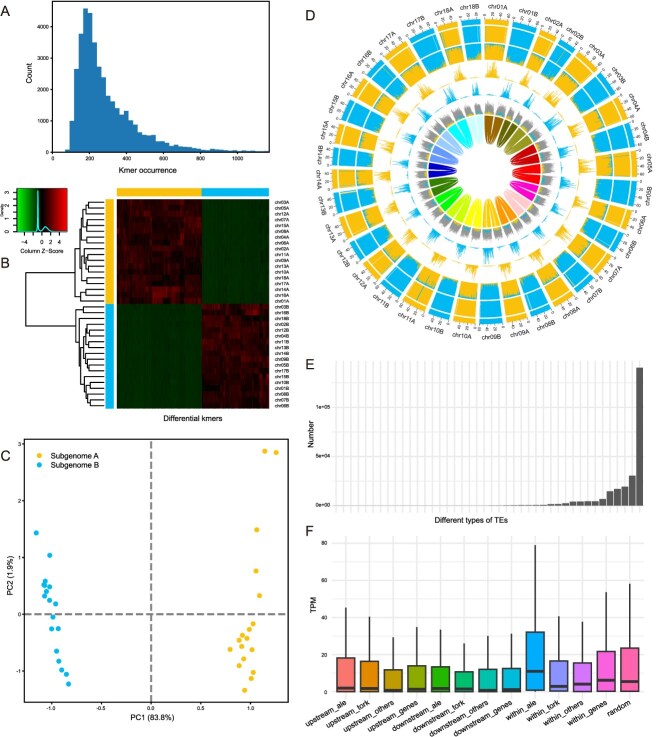
Subgenome phasing and characterization of the *F. albivenis* genome. (A) Histogram showing the occurrence frequency of all 15-mers in the genome. (B) Heatmap of unsupervised hierarchical clustering, where the horizontal color bar at the top indicates the subgenome specificity of the *k*-mers, and the vertical color bar on the left shows the subgenome assignment of the chromosomes. The heatmap itself represents the Z-scaled relative abundance of *k*-mers. (C) PCA plot showing the distribution of 15-mers in two subgenomes (Subgenome A and Subgenome B). Principal Component 1 (PC1) and Principal Component 2 (PC2) explain 83.8% and 19.9% of the total variance, respectively. Yellow and blue dots represent the two different subgenomes, indicating clear separation. (D) Circular genome plot illustrating the distribution of *k*-mers across chromosomes. From outer to inner rings (1–7): (1) subgenome assignments determined by *k*-means clustering; (2) significant enrichment of subgenome-specific *k*-mers, where colors matching the subgenome indicate significant enrichment, and white areas indicate no significant enrichment; (3) normalized relative proportion of subgenome-specific *k*-mers; (4, 5) absolute counts of each subgenome-specific *k*-mer set; (6) density of LTR-RTs, with matching subgenome colors indicating significant enrichment of LTR-RTs to subgenome-specific *k*-mers, and gray indicating nonspecific LTR-RTs; (7) homoeologous blocks. All statistics (2–6) are calculated in 1-Mb sliding windows. (E) Bar chart depicting the distribution of various TEs specific to Subgenome A. Most TE types are found in low numbers, while a few are abundant, highlighting significant variation. Each bar, from left to right, represents the following TE types: Class_I/DIRS, Class_I/LTR/Ty1_copia/Bryco, Class_I/LTR/Ty3_gypsy/chromovirus/chromo-unclass, Class_I/LTR/Ty3_gypsy/chromovirus/Tcn1, Class_I/Penelope, Class_II/Subclass_2/Maverick, Class_I/LTR/Ty1_copia/Lyco, Class_I/LTR/Ty3_gypsy/non-chromovirus/non-chromo-outgroup, Class_I/LTR/Ty3_gypsy/non-chromovirus/OTA/Tat/TatIII, Class_I/LTR/Ty3_gypsy/chromovirus/chromo-outgroup, Class_I/LTR/Retrovirus, Class_I/LTR/Ty1_copia/Osser, Class_II/Subclass_2/Helitron, Class_I/LTR/Ty1_copia/Gymco-II, Class_I/LTR/Ty1_copia/Gymco-I, Class_I/LTR/Ty3_gypsy/chromovirus/Chlamyvir, Class_II/Subclass_1/TIR/Tc1_Mariner, Class_I/LTR/Ty1_copia/Ty1-outgroup, Class_I/LTR/Ty3_gypsy/non-chromovirus/OTA/Tat/Retand, Class_II/Subclass_1/TIR/EnSpm_CACTA, Class_II/Subclass_1/TIR/PIF_Harbinger, Class_II/Subclass_1/TIR/hAT, Class_II/Subclass_1/TIR/MuDR_Mutator, Class_I/LTR/Ty3_gypsy, Class_I/LINE, Class_I/LTR/Ty1_copia, Class_I/LTR/Ty3_gypsy/chromovirus/Galadriel, Class_I/pararetrovirus, Class_I/LTR/Ty1_copia/Ikeros, Class_I/LTR/Ty1_copia/Alesia, Class_I/LTR/Ty1_copia/SIRE, Class_I/LTR/Ty3_gypsy/chromovirus/CRM, Class_I/LTR/Ty1_copia/TAR, Class_I/LTR/Ty1_copia/Angela, Class_I/LTR/Ty3_gypsy/chromovirus/Reina, Class_I/LTR/Ty1_copia/Bianca, Class_I/LTR/Ty1_copia/Ivana, Class_I/LTR/Ty3_gypsy/non-chromovirus/OTA/Tat/Ogre, Class_I/LTR/Ty1_copia/Tork, Class_I/LTR/Ty3_gypsy/chromovirus/Tekay, Class_I/LTR/Ty1_copia/Ale, Class_I/LTR/Ty3_gypsy/non-chromovirus/OTA/Athila. (F) Box plot showing the distribution of transcript expression (TPM) across various genomic regions, including 2 kb upstream of genes, within genes, and 2 kb downstream of genes. Each box represents the expression distribution in a specific region. ‘Others’ refers to all TEs except ale and tork; ‘genes’ refers to all TEs; ‘random’ refers to 500 genes randomly selected from the genome.

**Figure 4:**
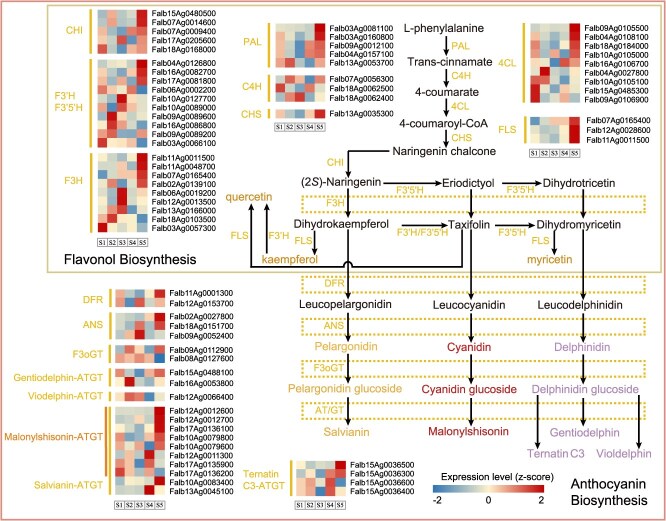
The metabolic pathways of flavonol and anthocyanin. The heatmap indicate the gene expression values (in normalized TPMs) at samples 1–5. Genes encode the following enzymes: PAL: phenylalanine ammonia-lyase; C4H: cinnamate-4-hydroxylase; 4CL: 4-coumarate CoA ligase; CHS: chalcone synthase; CHI: chalcone isomerase; F3H: flavanone 3-hydroxylase; F3’H: flavonoid 3-hydroxylase; F3’5’H: flavonoid 3,5-hydroxylase; FLS: flavonol synthase; DFR: dihydroflavonol reductase; ANS: anthocyanidin synthase; AT: acyltransferase; F3oGT: flavonol-3-O-glucosyl transferase; GT: glucosyltransferase.

### Phylogenetics and genome comparison

A phylogenetic tree was constructed including genomes from 16 species within the Lamiales order, one from the Solanales order, one from the Gentianales, one from the Garryales, and one outgroup (grape), along with subgenomes A and B of *F. albivenis*. The tree was based on 1171 orthogroups, with each orthogroup containing single-copy genes from at least 90.9% of the species analyzed. Phylogenetic analysis indicates that subgenomes A and B diverged between 1 and 2.2 million years ago (95% CI) (Fig. 2A). These subgenomes are most closely related to *Strobilanthes*, with divergence between these genera starting ~17.4–26.2 million years ago. Subgenomes A and B exhibit roughly equal numbers of expanded and contracted gene families, mostly involving the same families. For instance, in subgenome A, we identified 623 expanded, 481 contracted, 124 rapidly evolving, demonstrating significant dynamic changes in gene family composition. Interestingly, significant expansions have been observed in the families associated with terpene and terpenoid biosynthetic and metabolic processes, as well as flavonol biosynthetic and metabolic processes (Supplementary Table S8–S9).

*Fittonia albivenis* exhibits a 1:1 orthologous relationship with closely related species *Strobilanthes cusia* and *Andrographis paniculata* (Fig. 2B, Supplementary Fig. S9), as determined using the SOI software [25], indicating that no independent genome duplication events occurred in *F. albivenis* following its divergence from these genomes. The collinearity analysis between subgenomes A and B of *F. albivenis* demonstrates well-preserved gene order and strong synteny (Fig. 2C and D), despite the presence of numerous structural variations. A total of 281 inversions, 10 617 translocations, and 18 985 duplications were identified, affecting regions of 93.58, 52.15, and 80.72 Mb, respectively, with the largest structural variation located on chromosome 3 (Fig. 2D, Supplementary Table S10). In addition to these structural variations, we identified further genomic changes including 5.82 Mb of insertions, 9.00 Mb of deletions, 98.31 Mb of single nucleotide polymorphisms (SNPs), 2.20 Mb of copy number gains, and 5.87 Mb of copy number losses, showcasing the dynamic genomic landscape and evolutionary pressures acting on these subgenomes.

### Leaf coloration genes in *F. albivenis*

To delve into the molecular regulatory mechanisms underlying leaf color formation, we reconstructed the pigment metabolic pathways (flavonol, anthocyanin, chlorophyll, carotenoid, and xanthophyll) potentially linked to leaf coloration (Fig. 4 and Supplementary Fig. S10), based on our high-quality *F. albivenis* A genome. A total of 278 enzymatic genes were identified, including 58 genes involved in chlorophyll biosynthesis pathways, 99 genes from 30 enzymatic families related to carotenoid and xanthophyll biosynthesis, and 121 genes associated with flavonol and anthocyanin biosynthesis. It was discovered that gene duplications significantly contributed to the expansions of gene families in pigment-related pathways, with 60.34, 91.74, and 77.78% of genes in the chlorophyll, flavonol/anthocyanin, and carotenoid/xanthophyll pathways, respectively, being duplicated (Supplementary Table S11). Notably, whole-genome duplication (WGD) events were the predominant driver of these expansions, accounting for 25.86%, 33.06%, and 36.36% of genes in the chlorophyll, flavonol/anthocyanin, and carotenoid/xanthophyll pathways, respectively. Tandem duplication (TD), on the other hand, also played a significant role in amplifying genes in the flavonol/anthocyanin pathway, contributing ~32.23%. Interestingly, gene expression analysis revealed that genes linked to proximal duplication (PD) are characterized by the lowest expression levels (Supplementary Fig. S11).

### Subgenome-specific LTR-RT identification

Employing SubPhaser, we identified 14 658 and 11 635 subgenome-specific *k*-mer sequences from genomes A and B, respectively (Fig. 3A). These subgenome-specific *k*-mers were adequate for phasing the two genomes. These subgenome-specific *k*-mers collectively mapped to 255 672 TE sequences. Further classification of these TEs revealed that >99.3% of the subgenome-specific amplified TEs (254031) are LTR-retrotransposons (LTR-RTs), mirroring discoveries in other species such as wheat and mung bean [7,11]. These subgenome-specific LTR-RTs commenced their independent expansion ~2 million years ago (Supplementary Fig. S12), suggesting that the two genomes began diverging independently at that time. Subsequent recent hybridization events led to the formation of a new hybrid individual, aligning with divergence times inferred from phylogenetic analysis (Fig. 2A).

In the class of LTR-RTs, the subgroup Class_I/LTR/Ty3_gypsy/non-chromovirus/OTA/Athila represents the most abundant type, comprising 140 400 instances that account for 54.9% of the total LTR-RTs. This is followed by Class_I/LTR/Ty1_copia/Ale, Class_I/LTR/Ty3_gypsy/chromovirus/Tekay, Class_I/LTR/Ty1_copia/Tork, and Class_I/LTR/Ty3_gypsy/non-chromovirus/OTA/Tat/Ogre, with counts of 30 533, 19 405, 17 057, and 14 662, respectively, making up 11.9%, 7.6%, 6.7%, and 5.7% of the total LTR-RTs (Fig. 3E). These TEs inserted into 1329 gene regions, with significant LTR insertions (*P* < 0.05) found in 20 of the 278 pigment-related genes in the chlorophyll, flavonol/anthocyanin, and carotenoid/xanthophyll pathways, suggesting a potential regulatory role of TE insertions in leaf color synthesis. Further analysis revealed that the expression levels of these 20 genes with LTR insertions were lower than those without (Supplementary Fig. S13), suggesting that LTR insertion affects the expression of these genes in pathway.

**Figure 5:**
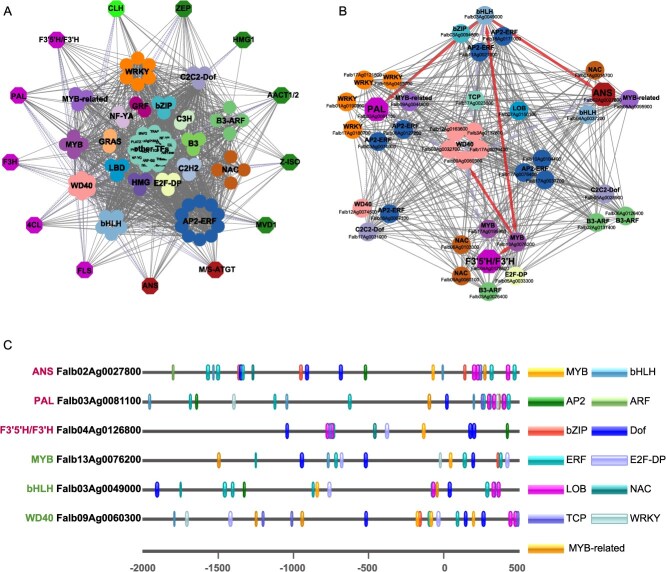
Identification of key TFs and transcriptional regulatory network associated with leaf color variation. (A) The gene regulatory network identified by coexpression analysis and TF binding site prediction. Magenta, Red, Green, and Dark green hexagons represent structural genes involved in flavonol, anthocyanin, chlorophyll degradation, and carotene/xanthophyll biosynthesis, respectively. Colored circles represent different families of transcription factors. (B) Subnetworks for the three key biosynthetic enzymes in the early and late stages of flavonol/anthocyanin synthesis (PAL, F3’5’H/F3’H, and ANS). (C) DNA-binding sites of promoter sequences of ANS, PAL, F3’5’H/F3’H, MYB, bHLH, and WD40 genes.

**Figure 6:**
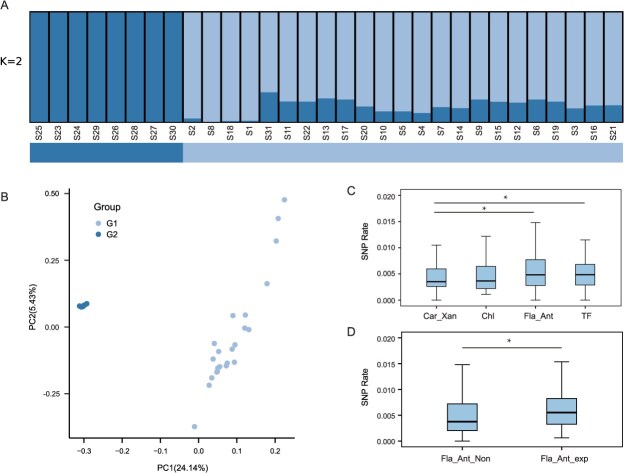
Population structure and SNP rate analysis. (A) Population structure inferred by STRUCTURE analysis for K = 2. Each individual is represented by a single vertical bar divided into segments representing the estimated membership proportions in each of the K clusters. (B) PCA of the genetic variation. Each point represents an individual and is colored according to the group assignment. (C) Box plot of SNP rate between various categories of genes: Car_Xan (Carotenoid/Xanthophyll), Chl (Chlorophyll), and Fla_Ant (Flavonol/Anthocyanin). Each box represents the interquartile range, the line within each box marks the median, and whiskers extend to the minimum and maximum values within 1.5 times the interquartile range. Significant differences in SNP rates between groups are indicated by asterisks (^*^P < 0.05). (D) Box plot of SNP rates of genes in the Flavonol/Anthocyanin pathway. Fla_Ant_exp represents genes with an expression value TPM ≥ 1 in at least one of the five samples, while Fla_Ant_Non represents genes with no expression (TPM < 1) in the Flavonol/Anthocyanin pathway. Significant differences in SNP rates between these two groups are indicated by an asterisk (^*^P < 0.05). Significance analysis was conducted using the wilcox.test function in R software.

We subsequently explored whether various classes of TEs exhibit preferential insertion into specific gene regions. Our findings indicated that despite having the highest number of insertions, Class_I/LTR/Ty3_gypsy/non-chromovirus/OTA/Athila did not display the highest insertion frequency in any specific gene region, including upstream, downstream, or within genes. Instead, Class_I/LTR/Ty1_copia/Ale and Class_I/LTR/Ty1_copia/Tork exhibited the most insertions. To further examine the impact of these different classes of TEs on gene expression, we categorized the TEs into three groups for statistical analysis: TE_Ale, TE_Tork, and TE_others. TE_Ale was found to insert into 188, 108, and 149 genes upstream, within, and downstream, respectively. TE_Tork inserted into 132, 58, and 135 genes in these regions, and TE_others into 254, 171, and 270 genes, respectively. Interestingly, we observed that insertions by TE_Ale in gene upstream and downstream regions had a lesser suppressive effect on gene expression compared to TE_Tork and TE_others (transcripts per million (TPM): TE_Ale > TE_Tork > TE_others) (Fig. 3F). GO enrichment analysis revealed that the insertion of these three types of TEs had no significant impact on any specific functional gene categories (Supplementary Table S12–S23).

### Candidate genes regulating pigment synthesis

To obtain further insights into the regulation of pigment-related pathways, we conducted RNA-seq and metabolomic profiling on leaves from five differentially colored samples (S1–S5, Supplementary Fig. S14). An average output of 7.77 Gb of clean bases per sample was obtained, with an average mapping rate of 91.59% (Supplementary Table S24). Cluster analysis of transcriptome profiles grouped the greenish leaf samples S1–S3 together (Supplementary Fig. S15). Differential expression analysis identified 5357 differentially expressed genes (DEGs), including 520 transcription factors (TFs). These DEGs were grouped into 14 clusters (R1–R14) based on similarity of expression patterns identified by the *k*-means algorithm, with each cluster containing 174–1185 genes (Supplementary Fig. S16). These gene clusters exhibited significant distinct expression patterns across differently colored samples. For instance, genes in R3 demonstrated a gradual increase in expression from S1 to S4, reaching a sharp peak at S5 (Supplementary Fig. S16), potentially playing a crucial role in mediating the color variation. Detailed analysis revealed 13 structural genes involved in pigment-related pathways for R3, including seven genes involved in flavonol/anthocyanin biosynthesis pathway, one gene associated with the chlorophyll degradation pathway, and five genes connected to carotene/xanthophyll biosynthesis pathway. Additionally, we discovered a total of 87 TFs for this cluster, mostly members of AP2-ERF, WRKY, WD40, and bHLH families.

Metabolomic analysis identified a total of 1068 annotated metabolites. In agreement with the transcriptome analysis, the samples were divisible into two groups—those with greenish leaves (S1–S3) and those with reddish leaves (S4–S5)—based on the expression patterns observed in the metabolome (Supplementary Fig. S17). Seven hundred thirty-seven compounds were found to be differentially expressed, with the majority being implicated in amino acid and derivatives (131 compounds), flavonoids (128 compounds), and carbohydrates and its derivatives (82 compounds). Clustering analysis categorized these differentially expressed metabolites (DEMs) into 14 clusters, designated as M1–M14, with each cluster containing 20–113 metabolites (Supplementary Fig. S18). Notably, the expression patterns of compounds within cluster M3 demonstrated a significant correlation with the RNA expression patterns observed in cluster R3, with a Pearson’s correlation coefficient of 0.97 and a *P*-value of 0.005. This alignment suggests that these metabolites within M3 could potentially play a pivotal role in the pigmentation variations observed in the leaves. Detailed analysis manifested that genes in involved flavonols/anthocyanins pathway were predominantly overexpressed in M3 (20 out of 57 compounds), followed by genes in amino acid and its derivatives pathway (14 compounds). The flavonol/anthocyanin metabolites comprised 10 flavones and flavonols, 4 isoflavonoids, 3 anthocyanins, 2 chalcones and dihydrochalcones, and 1 flavanone. Thus, we propose that the red-leaf phenotype may have been characterized and driven by flavonol/anthocyanin biosynthesis pathway, consistent with the gene expression patterns in these specific metabolic pathways.

To delineate the regulatory functions of key TFs in pigment biosynthesis pathways, we established a network based on the coexpression analysis between genes in pathway and TFs in R3, as well as flavonol/anthocyanin metabolites in M3. This analysis revealed 5355 correlations, including 909 TF–gene pairs in pathway and 179 metabolite–gene pairs in pathway (Supplementary Fig. S19). By correlating TF binding sites and the coexpression network, we reconstructed a regulatory network comprising 1259 pairs from 87 TFs and 13 genes, of which 243 were TF–gene pairs (Fig. 5A). Among those TFs, most members belonged to the AP2-ERF (10 genes) family, followed by WD40 (6 genes), WRKY (6 genes), and bHLH (5 genes). We identified that genes within multiple-pigment biosynthetic pathways could be concurrently regulated by the same TFs. For instance, an AP2-ERF family member, *Falb10Ag0104400*, could simultaneously regulate 11 enzymatic genes, including seven genes involved in flavonol/anthocyanin biosynthesis pathway (PAL, F3’5’H/F3’H, 4CL, F3H, FLS, ANS, and M/S-ATGT), three genes connected to carotene/xanthophyll biosynthesis pathway (MVD1, Z-ISO, and ZPE), and one gene associated with the chlorophyll degradation pathway (CLH) (Fig. 5A). A member of ZEP (*Falb09Ag0060600*), the key biosynthetic enzyme in xanthophyll biosynthesis pathway, was found to be regulated by the most regulators (25 TFs), mostly AP2-ERF (7 genes), WD40 (6 genes), and B3-ARF (3 genes). PAL, F3’5’H/F3’H, and ANS represent the key biosynthetic enzymes in the early and late stages of flavonol/anthocyanin synthesis pathway, respectively. We found that these three enzymatic genes are concurrently regulated by TFs including AP2-ERF (3 genes), WD40 (5 genes), LOB, and TCP, while also possessing their own distinct regulatory factors (Fig. 5B). In addition, we could infer that these three enzymatic genes may be regulated by the MYB-bHLH-WD40 (MBW) transcriptional activator complex derived from TF genes MYB *Falb13Ag0076200*, bHLH *Falb03Ag0049000*, and WD40 *Falb09Ag0060300*. *Cis*-element analysis revealed that the promoter regions of MYB, PAL, and ANS genes all contain bHLH binding sites, while F3’5’H/F3’H, bHLH, and WD40 genes possess MYB binding sites (Fig. 5C). This finding supports the regulatory role of the MBW complex in these three enzymatic genes.

### Genetic variations in candidate genes

We collected data from 31 individuals of *F. albivenis*, totaling 1 Tb, to investigate genetic variations in candidate genes, with an average of ~32.3 Gb per individual (Supplementary Table S25). Using genome assembly A as a reference, we mapped these data onto the genome, finding that >89% of reads aligned to the genome, with an average base alignment of 34.3 Gb and an average coverage depth exceeding 30× (Supplementary Table S22). After stringent quality control, we identified 5 373 038 high-quality SNPs.

Utilizing unlinked SNPs, admixture analysis determined that *K* = 2 is the optimal number of genetic groups to explain the structure among the 31 individuals (Fig. 6A). Principal component analysis (PCA) revealed similar groupings as those determined by admixture (Fig. 6B). In contrast to transcriptomic and metabolomic outcomes, population structure appears to be unrelated to leaf coloration. The five samples, which were divided into two groups based on transcriptomic and metabolomic data, did not align with the expected two groups corresponding to population structure (Fig. 6A). Genetic diversity metrics indicated that the genetic diversity in group 1 and group 2 was 0.0193 and 0.0099, respectively, with fixation index (*F*st) between the two groups of 0.0324.

Subsequently, we assessed gene mutations in the pathways responsible for the synthesis of chlorophyll, carotene/xanthophyll, and flavonol/anthocyanin, as well as their putative regulators. The mutation rate of genes in the flavonol/anthocyanin pathway was marginally higher than that of the others (Fig. 6C). Furthermore, analysis of the mutation rates among 87 candidate regulatory genes revealed their mutation rates was slightly lower than that of flavonol/anthocyanin synthesis pathway genes, but significantly higher than that of the carotene/xanthophyll synthesis pathway genes (Fig. 6C). Further analysis of the mutation regions of the genes revealed that, for both genes in pathway and candidate regulators, the mutation rates in the 3’UTR and 5’UTR regions were higher than those in the CDS and intron regions. Interestingly, the mutation rate in the CDS regions of genes in the flavonoid/anthocyanin synthesis pathway and candidate regulatory factors is significantly higher than that of the genes in the carotenoid/lutein and chlorophyll pathways (Supplementary Fig. S20A). Additionally, by categorizing the genes in the flavonoid/anthocyanin synthesis pathway into expressed and non-expressed groups, we observed that the mutation rate was significantly higher in the expressed genes (Fig. 6D). Further analysis of the genes expressed in the flavonoid/anthocyanin pathway revealed that 68.7% of these genes exhibited significant expression differences across samples with varying leaf colors. Moreover, the expression levels of these differentially expressed genes were significantly higher than those of genes without differential expression (Supplementary Fig. S20B). Therefore, mutations in genes of the flavonoid/anthocyanin pathway may be one of the factors contributing to leaf color diversity. However, the precise regulatory mechanisms underlying these differences require further investigation using larger population samples.

## Discussion

*Fittonia*, originally distributed in the tropical regions of South America, is now extensively cultivated and introduced as an important ornamental plant in various countries due to its variable leaf coloration, aesthetically pleasing patterns, and strong adaptability. Here, we utilized a combined approach involving PacBio HiFi, ultralong ONT, and Hi-C sequencing on hybrid F1 individuals to obtain the first nearly complete, haplotype-phased reference genome of *F. albivenis*. This is also the first nearly complete genome within the *Acanthaceae* family. This high-quality genome lays the foundation for subsequent comprehensive investigations into gene functionality and advancements in molecular breeding.

Based on the haplotype-phased genome assembly, we identified a large number of subgenome-specific amplified TE sequences, the majority of which are LTR-RTs (99.3%), consistent with conclusions previously obtained from haplotype-phased and polyploid genomes [7]. However, the relationship between these specifically amplified sequences and gene expression, as well as their potential specific impacts on certain gene functions, remains unclear. Taking the *F. albivenis* genome as an example, we employed TEsorter [26] to classify and categorize these TEs in detail. Upon examining the relationship between gene expression and subgenomic-specific amplified sequences, we found that most of the amplified TEs did not appear within or near genes (within 2 kb). Those located near genes typically led to reduced gene expression, with only a few TEs having the opposite effect, such as the significant upregulation of gene expression by Ale-type TEs inserted into these genes. Generally, these TEs do not have significant specific effects on certain functional genes. The insertion of these TEs may exhibit a certain degree of randomness.

In the realms of botany and genetics, the genesis of leaf coloration has persistently captivated scientists. This interest not only stems from its significant ornamental appeal but also for its potential to reflect the plant’s adaptive strategies to environmental stresses [27]. Comprehending the molecular mechanisms underlying leaf color formation holds substantial theoretical and practical value for botanical research and cultivar enhancement. In recent years, advancements in high-throughput sequencing technologies and genomics have enabled researchers to unveil the gene networks regulating pigment synthesis [28–30]. Based on the chromosome-scale genome of *F. albivenis*, in combination with the functional mapping and expression profiles, we reconstructed pigment metabolic pathways associated with leaf coloration, including flavonol, anthocyanin, chlorophyll, carotenoid, and xanthophyll, identifying a total of 278 enzymatic genes. It is noteworthy that gene duplication events have significantly propelled the expansion of gene families involved in pigment-related pathways, particularly evident in WGD events. TD, known to enhance gene product dosage and accelerate metabolic flux for rate-limiting steps in specific biosynthetic pathways, were found to be enriched in specific gene families [31,32]. In *F. albivenis*, TD have substantially increased the proportion of enzyme genes for flavonol/anthocyanin synthesis, highlighting their critical role in shaping leaf color diversity [27]. Additionally, we also found that genes associated with PD exhibit the lowest expression levels, suggesting these genes might have acquired novel functions or been silenced during evolutionary processes.

Subsequent RNA-seq and metabolomic analyses unveiled notable differences in both transcriptomic and metabolic profiles among samples of varying colors. Cluster analysis partitioned the transcriptome and metabolome into 14 clusters (R1–R14; M1–M14), with genes in cluster R3 showing a progressive increase in expression from samples S1 to S4, peaking in S5, potentially playing a pivotal role in color variation. The metabolic expression pattern in cluster M3 was highly correlated with the RNA expression pattern in R3, with flavonol and anthocyanin pathway metabolites highly enriched in M3, indicating that the overexpression of these metabolites might be one of the determinative factors for the red leaf phenotype. Recent studies have extensively detailed the regulation of the MBW transcriptional activation complex in flavonoid synthesis across various plant species [27,33–35], with several studies affirming the pivotal function of MYB TFs in modulating CHS and ANS genes [36,37]. Analysis of our gene coexpression networks suggests that the MBW complex might directly regulate ANS, PAL, and F3’5’H/F3’H genes, thereby enhancing the accumulation of anthocyanins and flavonols during the leaf coloration of *F. albivenis*. Previous research has unveiled novel mechanisms by which WRKY TFs, in conjunction with the MBW complex, regulate the anthocyanin pigment pathway, leading to the proposed ‘MBWW quartet’ model [34]. In Asian red-skinned pears, PyERF3 was found to interact with PyMYB114 and PybHLH3 to co-regulate anthocyanin biosynthesis in fruits [35]. From our coexpression network, we could speculate that WRKY and AP2-ERF families, known to be responsible for biotic/abiotic stress responses [38], may also be the potential regulators in leaf pigment biosynthesis, and likely play vital roles in leaf color variation and patterning [39]. Furthermore, the ethylene response factor, ERF, has been recognized as a crucial direct regulator within our leaf color variation regulatory network. This observation leads us to postulate that ethylene might influence leaf coloration, offering insights for prospective studies. We also examined the mutation rates of these genes and found an interesting pattern: genes in the flavonol/anthocyanin pathway and TFs exhibited higher mutation rates. Among the top five genes with the highest mutation rates, one was an AP2-ERF gene, while the other four were all involved in this pathway. This suggests that mutations in this TF and flavonol/anthocyanin genes in pathway may be closely linked to the variation in leaf coloration of *F. albivenis*.

In conclusion, the first nearly complete and haplotype-phased genome assembly presented here is expected to provide a reference for the further understanding of *Fittonia* evolution and diversity. Furthermore, the comprehensive transcriptome and metabolome investigations provided an in-depth insight into leaf coloration for *F. albivenis*. Gene duplication and key TFs may play crucial roles in pigment synthesis pathways, particularly WGD event and key TFs such as AP2-ERF, WD40, WRKY, MYB, and bHLH, which are pivotal in regulating the expression of genes related to leaf coloration. These findings not only enrich our understanding of plant pigment metabolism and its regulatory mechanisms but also provide a crucial theoretical foundation for the enhancement and breeding of plant colors. Future research can further investigate the specific mechanisms of these regulatory factors and how to utilize this genetic information for the targeted improvement of plant coloration.

## Materials and methods

### Sample collection, library preparation, and sequencing

Young leaves of *F. albivenis* were collected, rapidly frozen in liquid nitrogen, and subsequently stored at −80°C. Genomic DNA extraction was performed utilizing a modified CTAB protocol [40].

The sequencing of the *F. albivenis* genome was conducted using four different methodologies. Initially, genomic DNA was quantified and evaluated for both integrity and purity. A total of 1 μg of DNA was then fragmented using Covaris ultrasonication to achieve fragments ranging from 200 to 400 bp. Libraries were constructed and subjected to paired-end sequencing on the DNBSEQ platform.

In the second approach, DNA integrity was confirmed using Femto Pulse, followed by library preparation with the Pacific Biosciences SMRTbell Express Template Prep Kit 2.0, with sequencing performed on the PacBio Sequel II platform. Additionally, DNA fragments that met quality control standards were used for library preparation with the Oxford Nanopore SQK-LSK109 kit, and sequencing was conducted using R9.4 flow cells on the PromethION sequencer (Oxford Nanopore Technologies, Oxford, UK). Finally, Hi-C libraries were prepared in accordance with the protocol outlined by Wang *et al*. [41] and sequenced on the DNBSEQ platform.

### Genome survey

We assessed the heterozygosity of the sample by performing a genome survey with GenomeScope [13]. To investigate the ploidy level of the genome, we used Smudgeplot [13], a tool that infers ploidy from HiFi sequencing data.

### *De novo* genome assembly

The genome assembly of *F. albivenis* was achieved through a combination of PacBio HiFi, Oxford Nanopore (ONT), and Hi-C reads. The initial step involved generating primary contigs using hifiasm v0.16.1-r375 with PacBio HiFi reads [14]. Chromosome-scale scaffolding was performed by aligning Hi-C reads to the primary assembly using Juicer v1.6 [15], followed by initial clustering with 3D-DNA v180922 [42] and manual inspection and refinement of misassemblies via Juicebox v1.11.08 [16]. This resulted in chromosome-scale scaffolds and unplaced contigs containing 100-bp gaps.

Gap closure was accomplished by assembling ONT reads with NextDenovo [17] and aligning the resulting ONT contigs to the chromosomal sequences using Unimap (https://github.com/lh3/unimap). Telomere repeats were extended by realigning HiFi reads to the ends of chromosomes. Organelle genomes were assembled separately using GetOrganelle [18].

The final assembly was polished through two rounds of NextPolish v1.3.1 [43] utilizing HiFi reads. Redundancies and potential contaminants were addressed by aligning unplaced contigs to the chromosomal and organellar sequences using redundans v0.13c [44], allowing for the identification and removal of haplotigs, organellar fragments, and rDNA fragments following manual verification. This hybrid approach, which integrated long reads, Hi-C scaffolding, and HiFi polishing, resulted in a high-quality, chromosome-scale reference genome assembly.

### Telomere and centromeric repeat sequences identification

Telomeres were identified by searching for the canonical telomeric repeat sequence ‘TTTAGGG/CCCTAAA’ within the genomic DNA, specifically targeting these known repeats. To detect conserved repeat motifs throughout the genome, the Tandem Repeats Finder algorithm v4.09 [45] was employed. For visualizing genomic karyotypes and detailing chromosomal features, karyoploteR v1.20.3 was applied [46].

### Repeat identification

TEs were *de novo* identified from the genome using EDTA v1.9.9 [19] with sensitive parameters (−sensitive 1 −anno 1), resulting in a comprehensive TE library. This library was then utilized in conjunction with RepeatMasker [20] to identify and annotate repetitive elements within the genome. Additionally, TEsorter v1.4.1 [26] was utilized for classifying LTR-RTs.

### Gene prediction and function annotation

The annotation of the *F. albivenis* genome was based on a combination of homologous proteins, transcript data, and *ab initio* predictions, creating a comprehensive gene map. Initially, homologous protein evidence was gathered from 283 692 nonredundant protein sequences across 10 plant species, including *A. paniculata*, *Avicennia marina*, *S. cusia*, *Buddleja alternifolia*, *Sesamum indicum*, *Antirrhinum majus*, *Jasminum sambac*, *Tectona grandis*, *Vitis vinifera*, and *Arabidopsis thaliana*.

For transcript evidence, pooled samples from roots, stems, and leaves were sequenced using Nanopore technology, yielding 10G of full-length cDNA reads. These reads were aligned to the genome using minimap2 v2.24-r1122 [21] and assembled with StringTie v1.3.5 [22]. Gene structures were annotated using PASA v2.4.1 [47], which identified full-length genes by comparing them to reference proteins.

Gene prediction was enhanced using AUGUSTUS v3.4.0 [48], which was trained specifically on the full-length gene set obtained earlier. The MAKER v2.31.9 [49] annotation pipeline further integrated *ab initio* predictions, transcript evidence, and homologous protein evidence to refine gene models. EvidenceModeler (EVM) v1.1.1 [50] was used to integrate and finalize these annotations, while TEsorter [26] masked TE protein domains. Genes with abnormal coding frames or lengths shorter than 50 amino acids were excluded from the final annotation.

ncRNA annotation was performed using tRNAScan-SE v1.3.1 [51], barrnap, and RfamScan [52].

Functional annotation of protein-coding genes employed three complementary strategies: alignment to the eggNOG v5.0 ortholog database using eggNOG-mapper v2.0.1 [53] to infer GO and KEGG pathways; similarity searching against protein databases such as SwissProt, TrEMBL, NR, and *A. thaliana* using diamond v0.9.24 [54] to identify best-matching orthologs; and domain architecture analysis through scanning with Hidden Markov Models in the InterPro database using InterProScan v5.27–66.0 [55]. This comprehensive approach provided extensive functional annotations, aligning the identified protein-coding genes with known sequences and conserved domain signatures.

### Assessment of genome completeness and protein-coding genes

The quality of the *F. albivenis* genome assembly was rigorously assessed using a tripartite approach to ensure accuracy and completeness. First, BUSCO v2.0.1 [23] was used to evaluate the presence and completeness of core conserved genes within the assembly, offering a measure of genomic completeness. Second, Merqury [24] assessed the assembly’s fidelity with a *k*-mer size of 19, providing insights into accuracy through *k*-mer-based metrics to identify potential sequencing errors. Finally, Hi-C data was mapped onto the assembled genome using Juicer, a critical step for detecting structural assembly errors and confirming the accuracy of the chromosome-scale organization and scaffold connections established during the assembly process.

### Genome comparison and subgenome-specific sequence identification

Genome assemblies were aligned using minimap2, which laid the groundwork for identifying syntenic regions and structural variations. Subsequently, SyRI v1.4 [56] was employed to comprehensively analyze syntenic regions and structural variations between closely related genomes. Once identified, the structural variations were visualized using Plotsr v0.5.4 [57]. For the analysis of subgenome-specific sequences, a *k*-mer-based approach was utilized via Subphaser [7,58,59]. Using *V. vinifera* as the outgroup, we classified gene duplications into five categories based on their genomic context: WGD, TD, PD (duplicates within 10 genes on the same chromosome), transposed duplications (TRD), and dispersed duplications (DSD; all other types not included in the above). Duplication events were identified using DupGen_finder [60]. The SOI software [25] was used to identify orthologous genes across different species.

### Phylogenetic analysis and estimation of species divergence times

We selected the longest protein-coding genes from 21 representative species across the Lamiales order and other related clades for orthologous gene inference using OrthoFinder2 v2.3.1 [61]. The resulting single-copy orthologs, present in at least 70% of species, were then used to construct a species tree with ASTRAL [62]. To estimate species divergence times, we performed a codon-based alignment of 188 single-copy orthologous genes and used the MCMCTree module of the PAML package [63]. Codon positions were assumed to have different substitution rates, and the molecular clock model applied was ‘independent rates’ (clock = 2). The nucleotide substitution model used was GTR. The first 500 000 iterations of the MCMC chain were discarded as burn-in, and every 100th iteration was sampled, totaling 100 000 samples. Divergence times were calibrated using data from *V. vinifera* and *Coffea canephora*, *S. indicum* and *Coffea canephora*, and *S. indicum* and *J. sambac*, obtained from TimeTree (https://timetree.org/). Additionally, we used CAFÉ [64] to detect expansions, contractions, and rapidly evolving gene families based on the time-calibrated phylogeny and 15 077 orthologous gene families.

### Transcriptomic analyses

The leaves from five differentially colored samples (S1–S5, Supplementary Fig. S10) with three biological replicates each were harvested and processed for RNA sequencing using the DNBSEQ-T7 platform. For preprocessing and quality control of RNA sequencing data, fastp v0.12.4 [65] was employed to clean the raw reads by removing any that were shorter than 60 bp, along with potential adapter sequences and low-quality reads. Following the preprocessing, salmon v1.6.0 [66] was used to quantify gene expression levels. Key options such as –validateMappings, which ensures accurate mapping by validating read alignments, and –numBootstraps 100, which provides statistical robustness through bootstrapping, were included to enhance the reliability of the expression estimates. Subsequent to quantification, the results were processed using tximport v1.22.0 [67]. Expression estimates were normalized to library size (TPM), and pairwise Pearson correlations were computed on the log_2_(TPM + 1) transformed data to evaluate replicate reproducibility. The samples of S1_3 and S5_3 were discarded as technical outliers due to the less correlations (R < 0.96). DEseq2 [68] was applied to identify the significant DEGs, with a log_2_ fold-change (FC) cut-off value of 1 and FDR ≤ 0.05.

### Metabolomic analyses

The metabolome of leaves from five samples (S1–S5) with three biological replicates each was evaluated using the Quasi-Targeted metabolomics method. Metabolite quantification was performed using a scheduled multiple reaction monitoring (MRM) method [69,70]. Metabolite annotation was conducted against a composite database integrating theoretical adducts from HMDB, LipidMaps, and KEGG. For quality control, ion peaks with >50% missing values per group were excluded, and zero values were imputed with half the minimum detected value. Metabolites scoring <30 (out of 60) were also removed. Positive and negative mode data were combined for PCA and (orthogonal) partial least-squares discriminant analysis ((O)PLS-DA) using the *ropls* package in R [33]. DEMs were defined by variable importance in projection (VIP) > 1 and *P* ≤ 0.05.

### Establishment of the coexpression network

To unveil the intricate genetic regulatory network of color variation, the R package cluster v.4.4.0 with the *k*-means method was used to analyze the coexpression patterns of transcriptome and metabolome. Correlation analysis between modules was performed using Mantel tests in R v4.4.0. Pearson’s correlation analysis was performed to identify the associations between DEGs and DEMs (r ≥ 0.87, *P* ≤ 0.05) using the Hmisc 4.1.0 package. The 2000-bp upstream and 500-bp downstream sequences of 5’ UTR of crucial genes were defined as the promoter regions, from which the *cis*-acting regulatory elements were identified with PlantTFDB v5.0 [71, 72]. The networks were visualized using Cytoscape v3.10.0 [73].

### Genome resequencing and SNP calling

The total genomic DNA from 31 individuals of *F. albivenis* was resequenced on the DNBSEQ platform to obtain >30×. Low-quality reads (≥50% bases with quality score <Q20) were removed to obtain clean data. Paired-end reads were aligned to the assembled *F. albivenis*-A reference genome using BWA with default settings. SNP calling was performed with Freebayes [74]. To reduce SNP calling bias, we applied filtering steps using VCFtools [75] and BCFtools [76], retaining only high-confidence SNPs. Filters included: (1) retention of bi-allelic SNPs located >5 bp from any indel and (2) treating genotypes with quality score <20 or depth <5 as missing. SNPs with >20% missing data or minor allele frequency (MAF) <5% were subsequently removed.

### Population structure analysis and calculation of population parameters

To reduce linkage disequilibrium, we pruned high-quality SNPs using PLINK [77] with the parameter ‘–indep-pairwise 50 10 0.58’, resulting in 377 820 independent SNPs. Population admixture was assessed using ADMIXTURE v1.3.0 [78] under varying ancestral clusters (K), based on the pruned dataset. PCA was performed with GCTA [79]. Visualization of results was carried out using CLUMPAK [80] and ggplot2 v3.4.2. Pairwise *F*_ST_ and nucleotide diversity (θπ) were calculated in ANGSD [81] over non-overlapping 2-kb windows based on sample allele frequency likelihoods. Gene mutation rates were estimated as SNP rate = SNP count/gene length.

### GO enrichment analysis

GO enrichment analysis was conducted using the R package clusterProfiler v4.2.2 [82]. First, we identified enriched GO terms with the enrichGO function. To reduce redundancy among these terms, we used the simplify function. We then assessed similarity between terms using the pairwise_termsim method with the Jaccard coefficient. Finally, the results were visualized using enrichplot v1.14.2 [83], which helped illustrate the relationships between significant GO terms in a network diagram.

## Supplementary Material

Web_Material_uhaf154

## Data Availability

The raw sequence data reported in this paper have been deposited in the Genome Sequence Archive in National Genomics Data Center, China National Center for Bioinformation/Beijing Institute of Genomics, Chinese Academy of Sciences (GSA: CRA017781) that are publicly accessible at https://ngdc.cncb.ac.cn/gsa. The whole-genome sequence data reported in this paper have been deposited in the Genome Warehouse in National Genomics Data Center, Beijing Institute of Genomics, Chinese Academy of Sciences/China National Center for Bioinformation, under accession number of GWHEUVN00000000.1 that is publicly accessible at https://ngdc.cncb.ac.cn/gwh.
