# Supplementary material for: A nearly complete haplotype-phased genome assembly of nerve plant (Fittonia albivenis) provides insights into leaf color evolution
Source: Hortic Res. 2025 Jun 26;12(9):uhaf154. doi: 10.1093/hr/uhaf154 (PMC12422009; doi:10.1093/hr/uhaf154)
Supplement: Web_Material_uhaf154 [file web_material_uhaf154.zip › Supplementary Figs.docx]

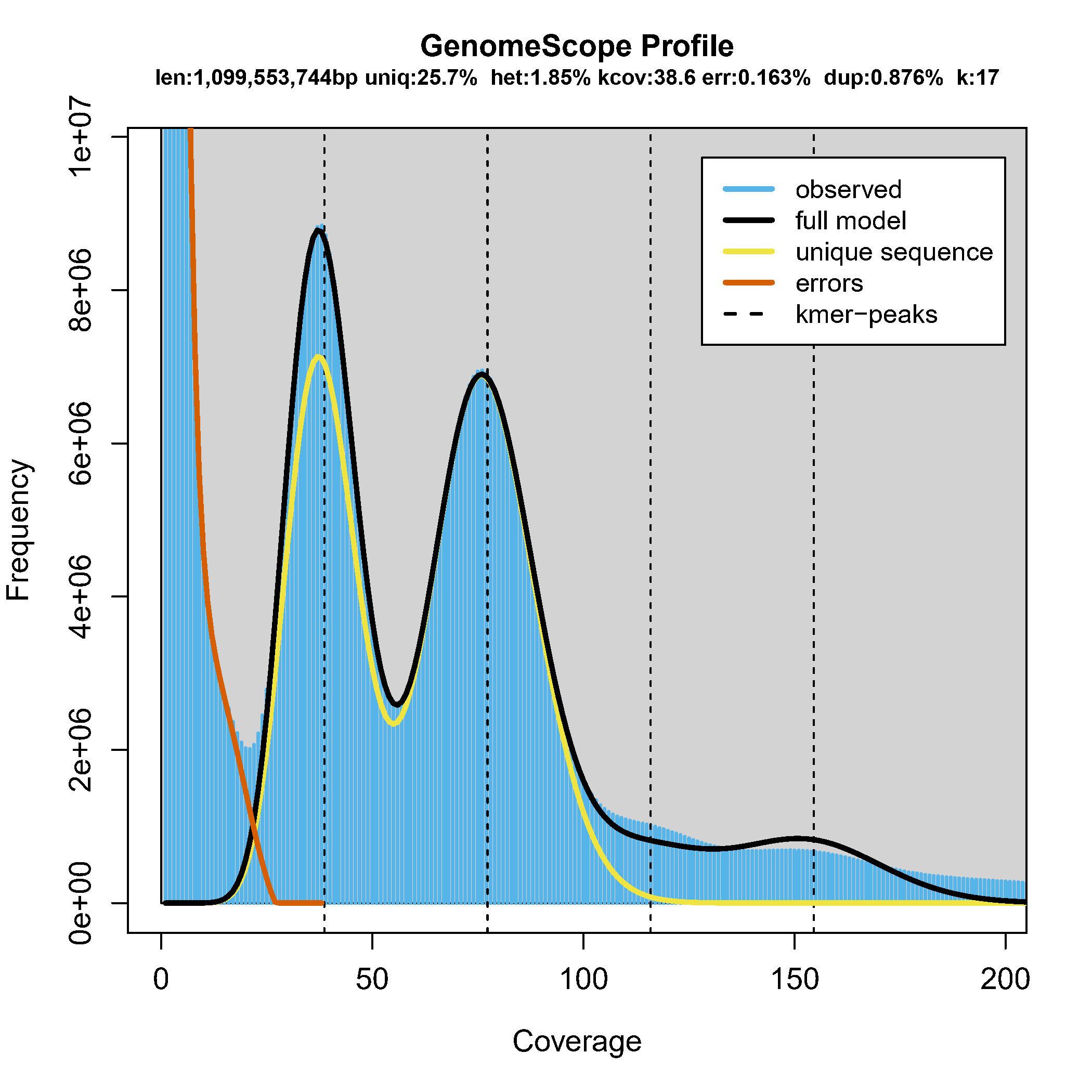


Supplementary Fig. 1 GenomeScope analysis based on 17-mer frequency distribution from DNBSEQ sequencing data. The blue histogram represents observed *k*-mer frequencies; the black line shows the fitted model. Yellow and orange curves indicate unique and erroneous k-mers, respectively.


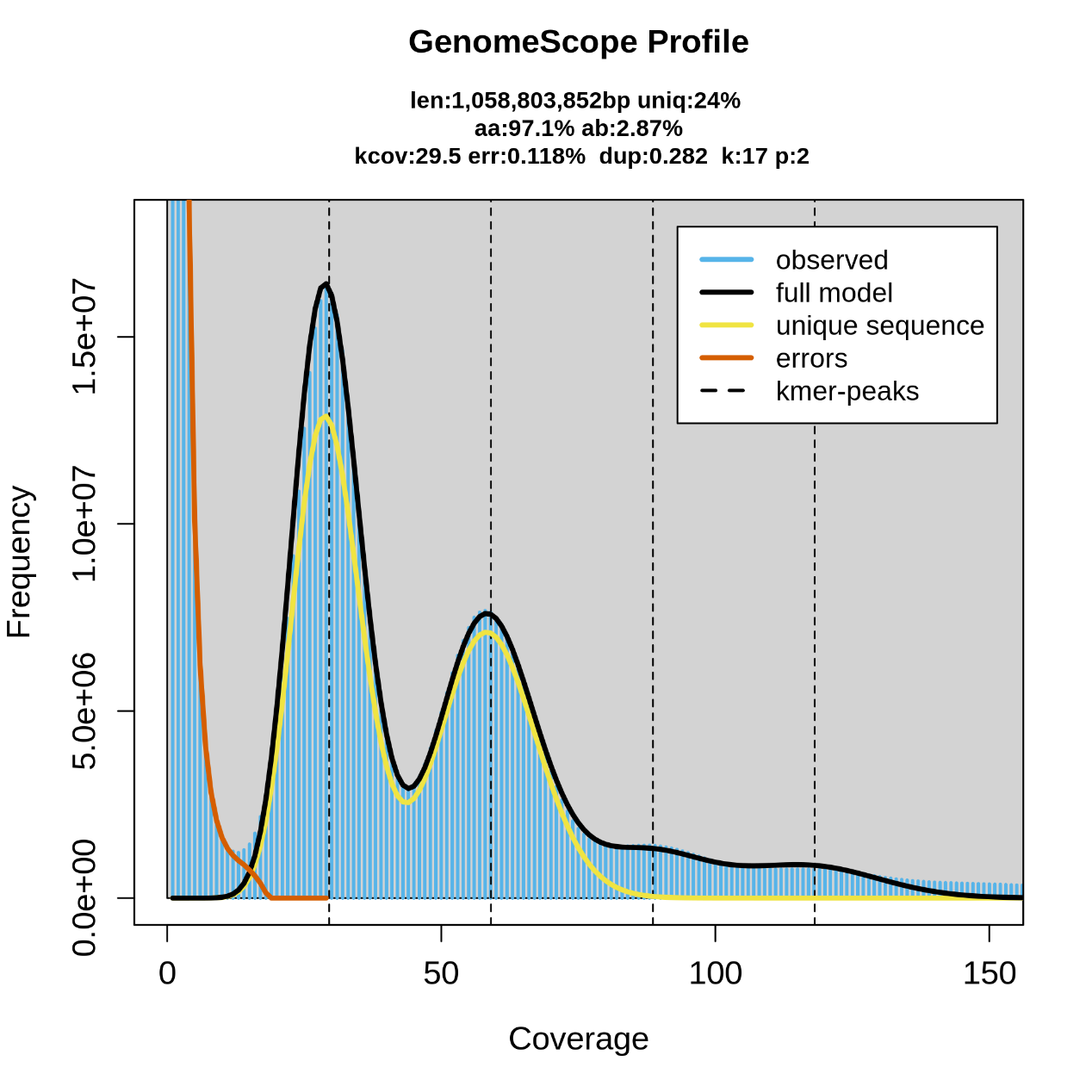


Supplementary Fig. 2 GenomeScope analysis based on 17-mer frequency distribution from HiFi sequencing data. The blue histogram represents observed *k*-mer frequencies; the black line shows the fitted model. Yellow and orange curves indicate unique and erroneous *k*-mers, respectively.


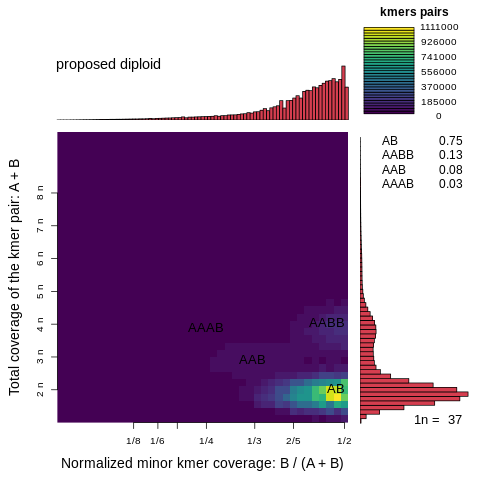
Supplementary Fig. 3 Smudgeplot analysis based on HiFi sequencing data. The plot shows the distribution of normalized minor *k*-mer coverage (B / (A + B)) against total *k*-mer pair coverage (A + B), supporting a diploid genome structure. Major *k*-mer pair types are labeled, with AB (0.75) being the predominant signal, consistent with diploidy.


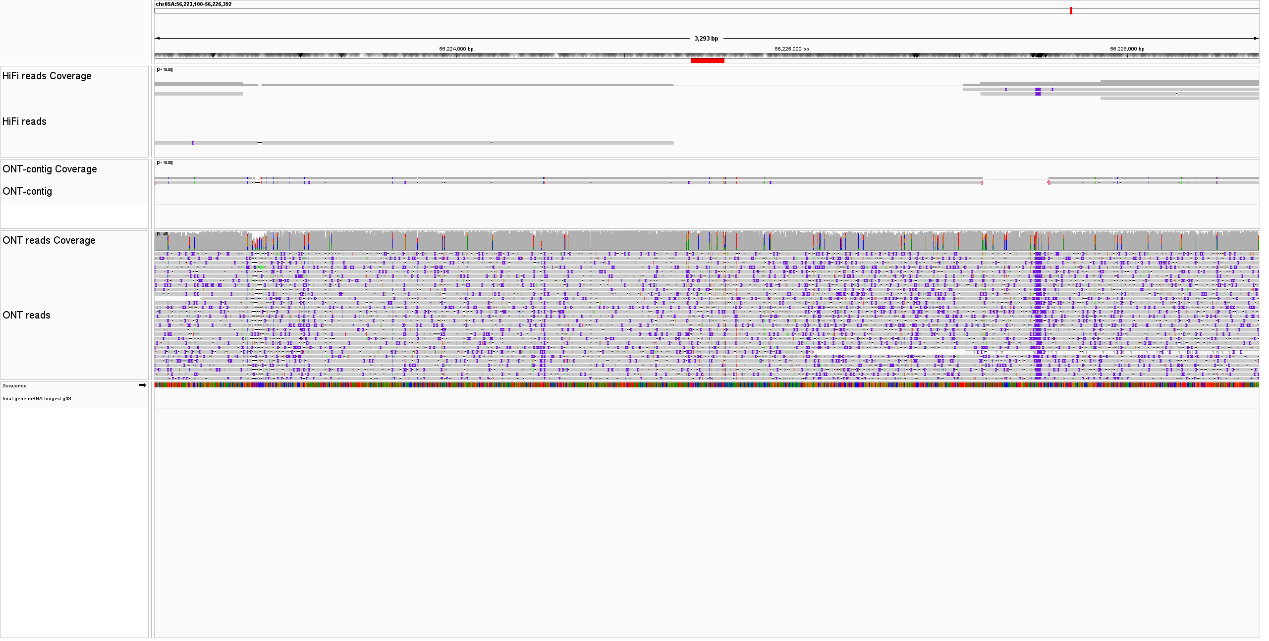


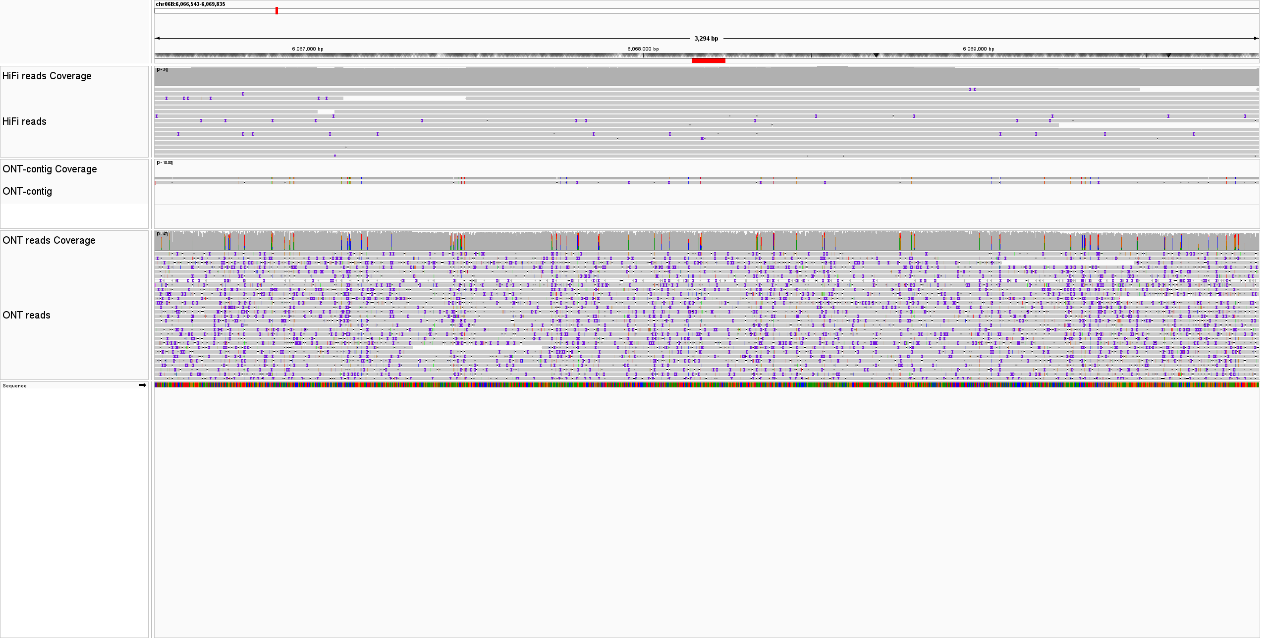


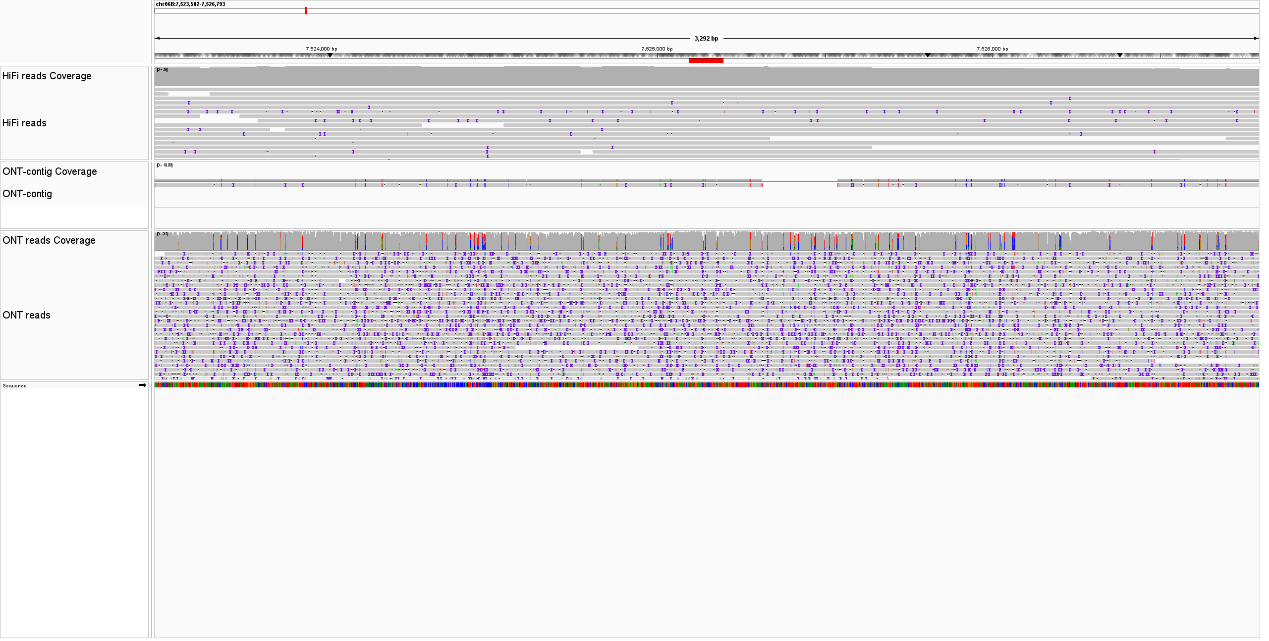


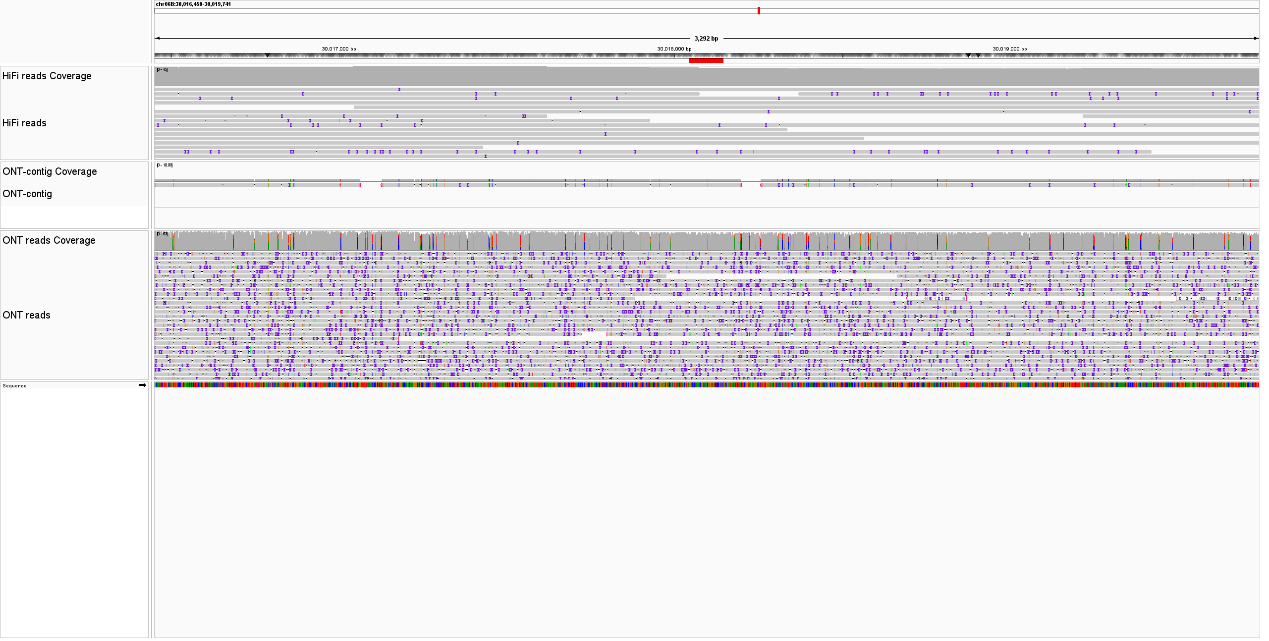


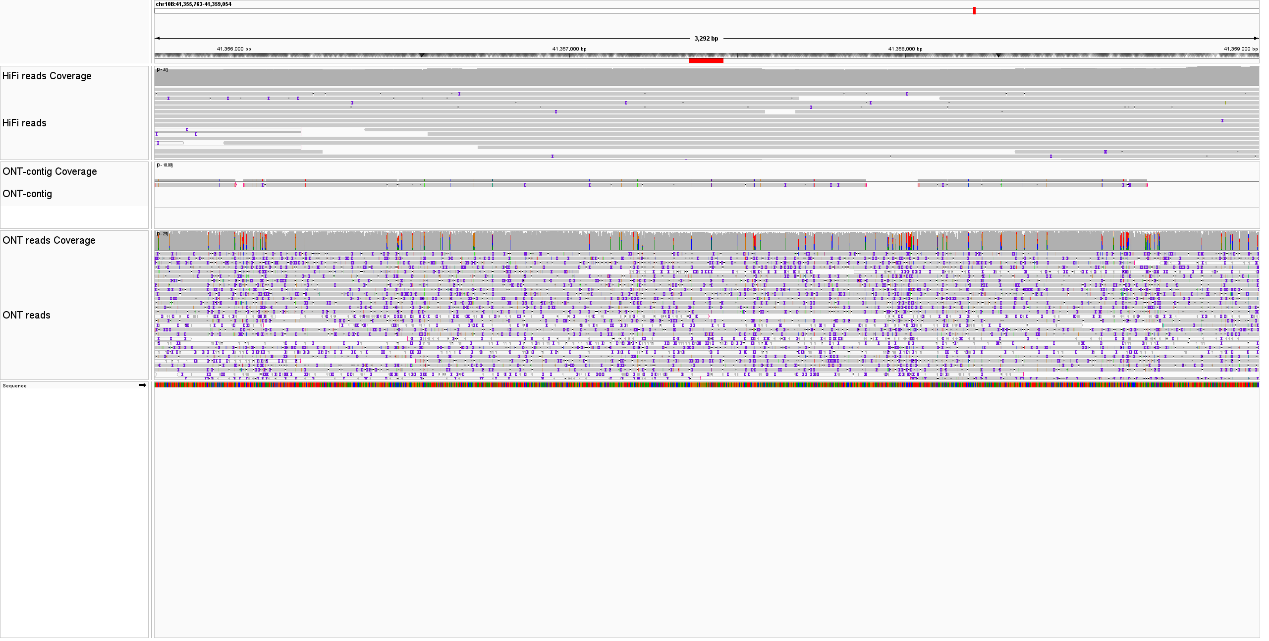


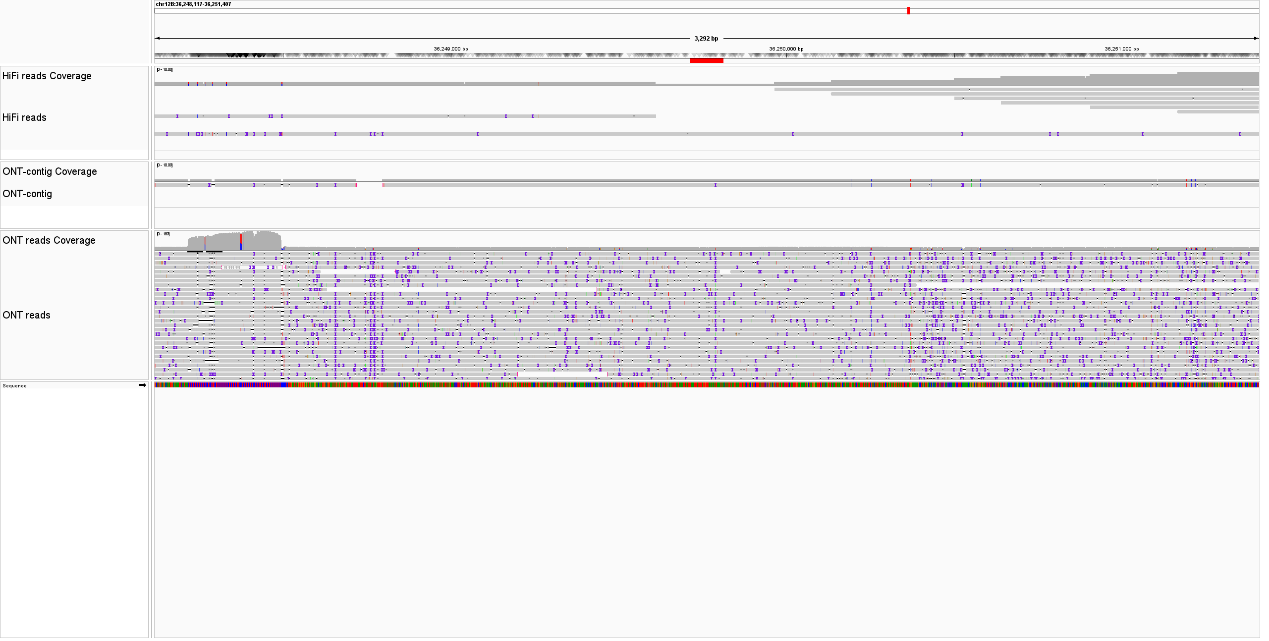


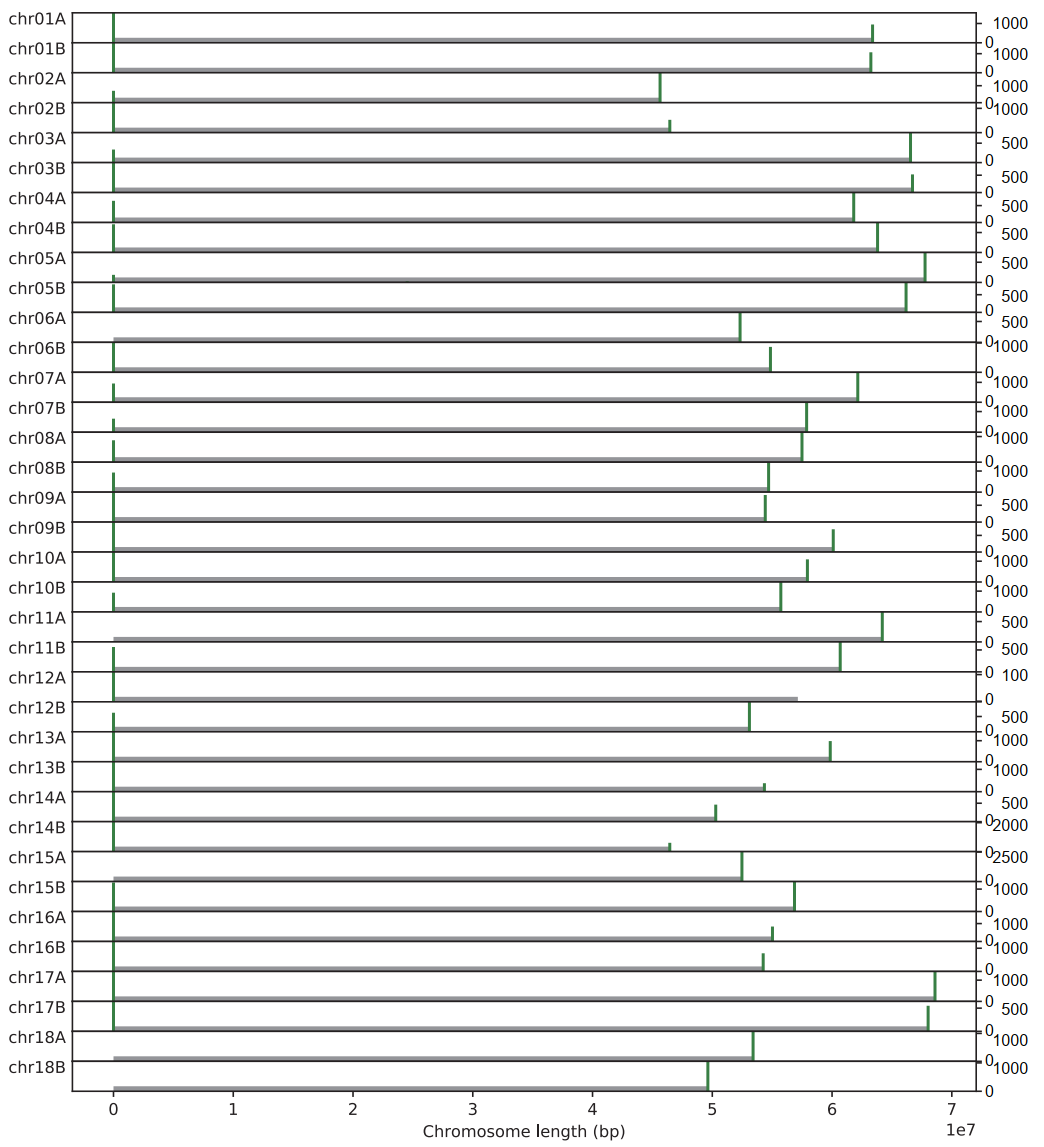
Supplementary Fig. 4 Integrative Genomics Viewer (IGV) screenshot showing the alignment of PacBio HiFi and ONT reads to the assembled genome. HiFi and ONT contigs, as well as individual reads, are displayed with coverage tracks.

Supplementary Fig. 5 Distribution of telomere positions along each chromosome based on the assembled genome. Chromosome lengths are shown on the x-axis, and telomere signal intensities are plotted on the right y-axis. Green bars indicate the positions and relative quantities of detected telomeric sequences across both A and B subgenomes.


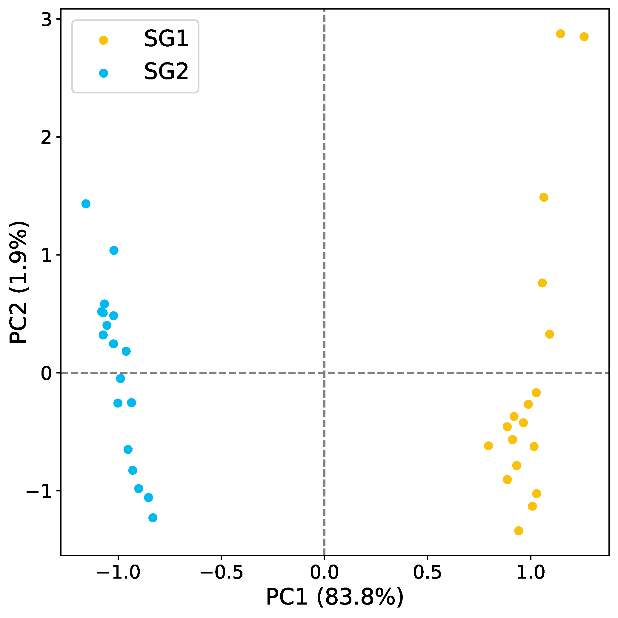

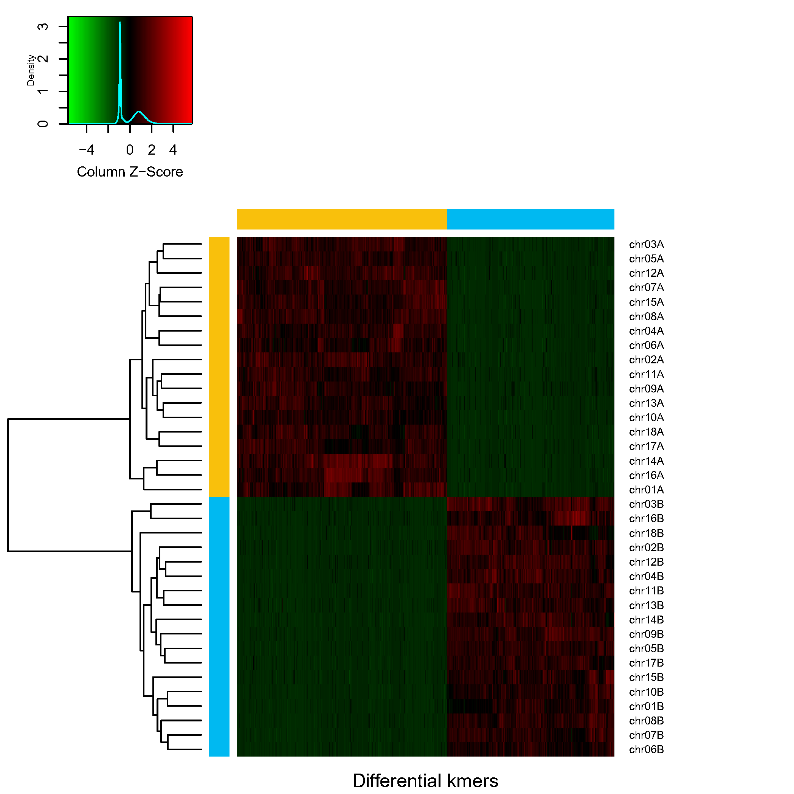


Supplementary Fig. 6 Subphaser analysis results based on differential *k*-mers. (Left) Heatmap showing the hierarchical clustering of chromosomes based on the abundance of differential *k*-mers, with two distinct subgenome groups (SG1 and SG2) indicated by color bars. (Right) Principal component analysis (PCA) plot further separating the two subgenome groups, with PC1 explaining 83.8% of the variance and PC2 explaining 1.9%.


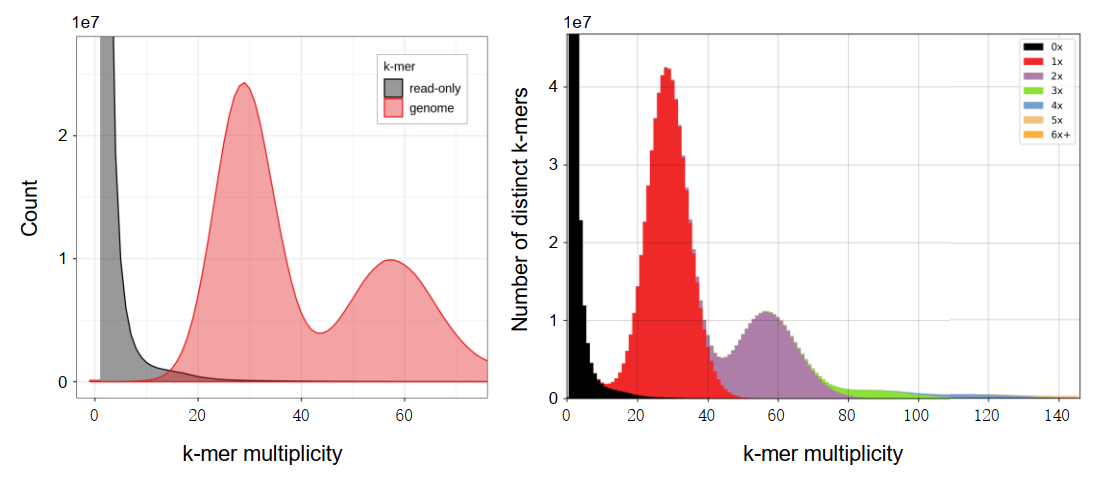


Supplementary Fig. 7 *K*-mer multiplicity analysis based on HiFi sequencing data. (Left) Distribution of *k*-mers comparing read-only *k*-mers (gray) and genome *k*-mers (red). (Right) Classification of distinct *k*-mers into different multiplicity levels.


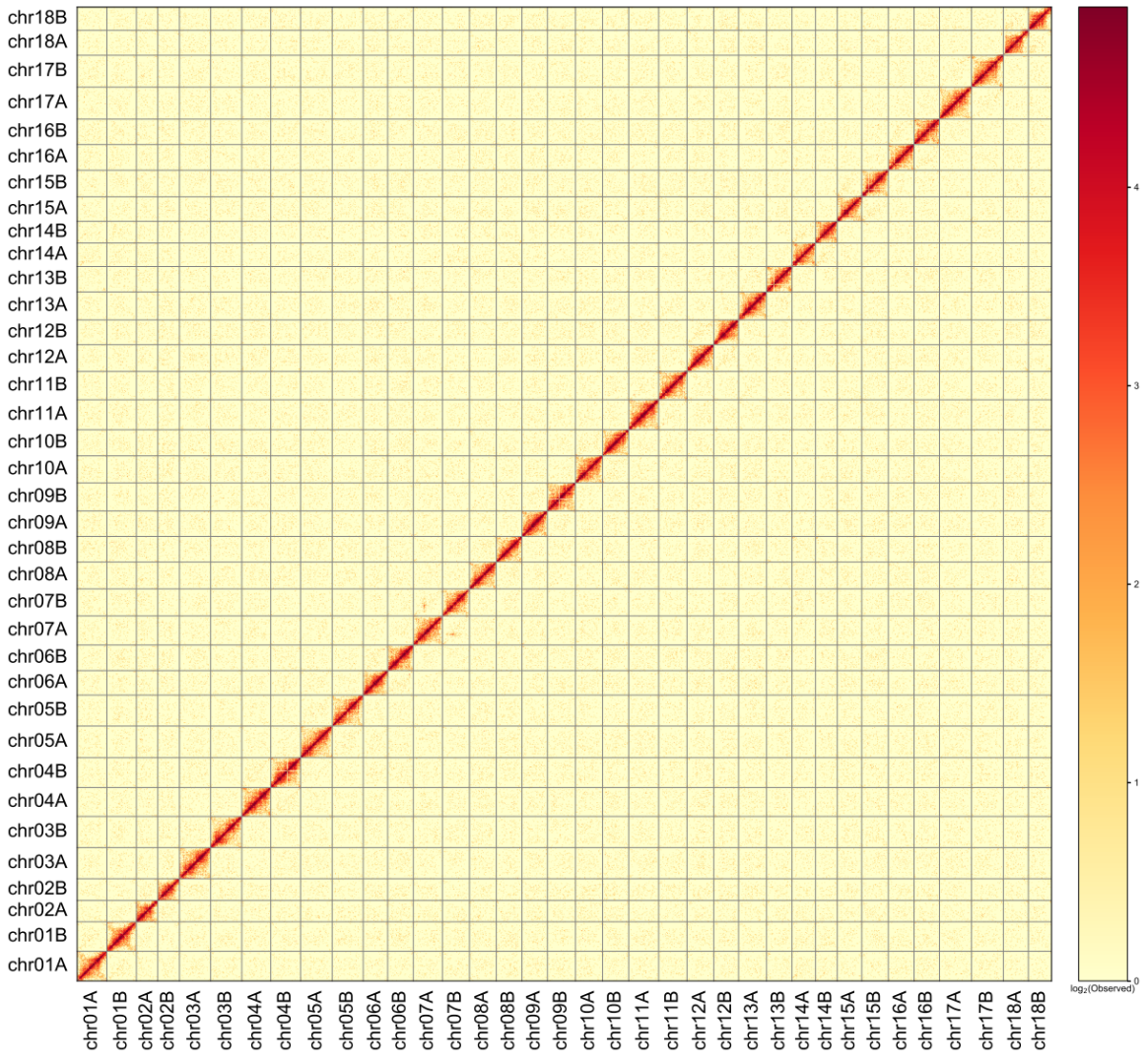


Supplementary Fig. 8 Hi-C contact heatmap of the assembled genome. Interaction frequencies are shown on a log-transformed color scale, with warmer colors indicating higher contact frequencies. Chromosomes are ordered and labeled along both axes, supporting the accuracy and continuity of the chromosome-level assembly.


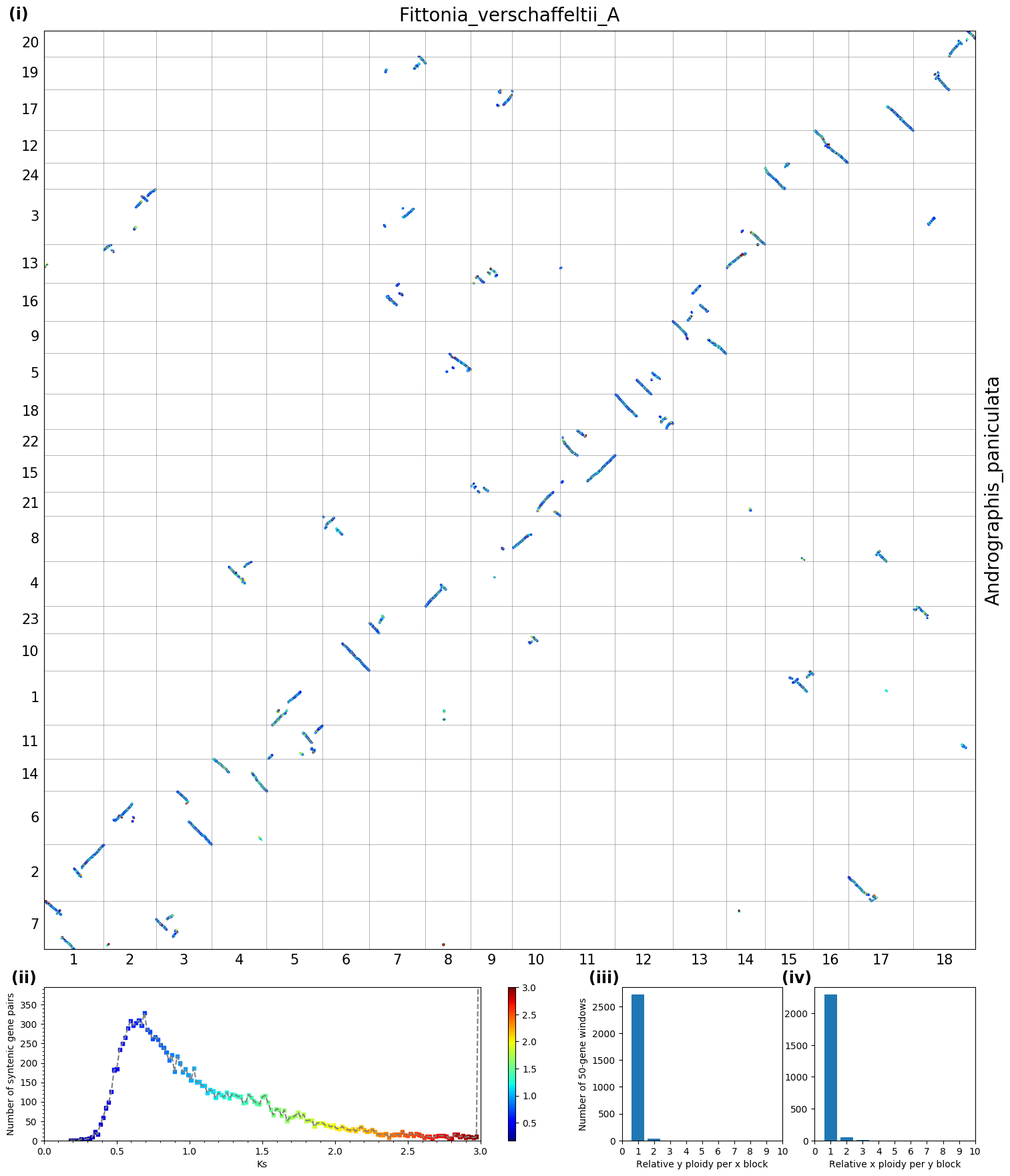
Supplementary Fig. 9 Comparative genomics and WGD analysis. (i) Syntenic dotplot between *Fittonia verschaffeltii* subgenome A and *Andrographis paniculata*. (ii) Distribution of synonymous substitution rates (*Ks*) for syntenic gene pairs, with a major peak indicating a recent WGD event. (iii–iv) Relative ploidy level distributions across genomic regions, showing genome-wide patterns of duplication and fractionation.


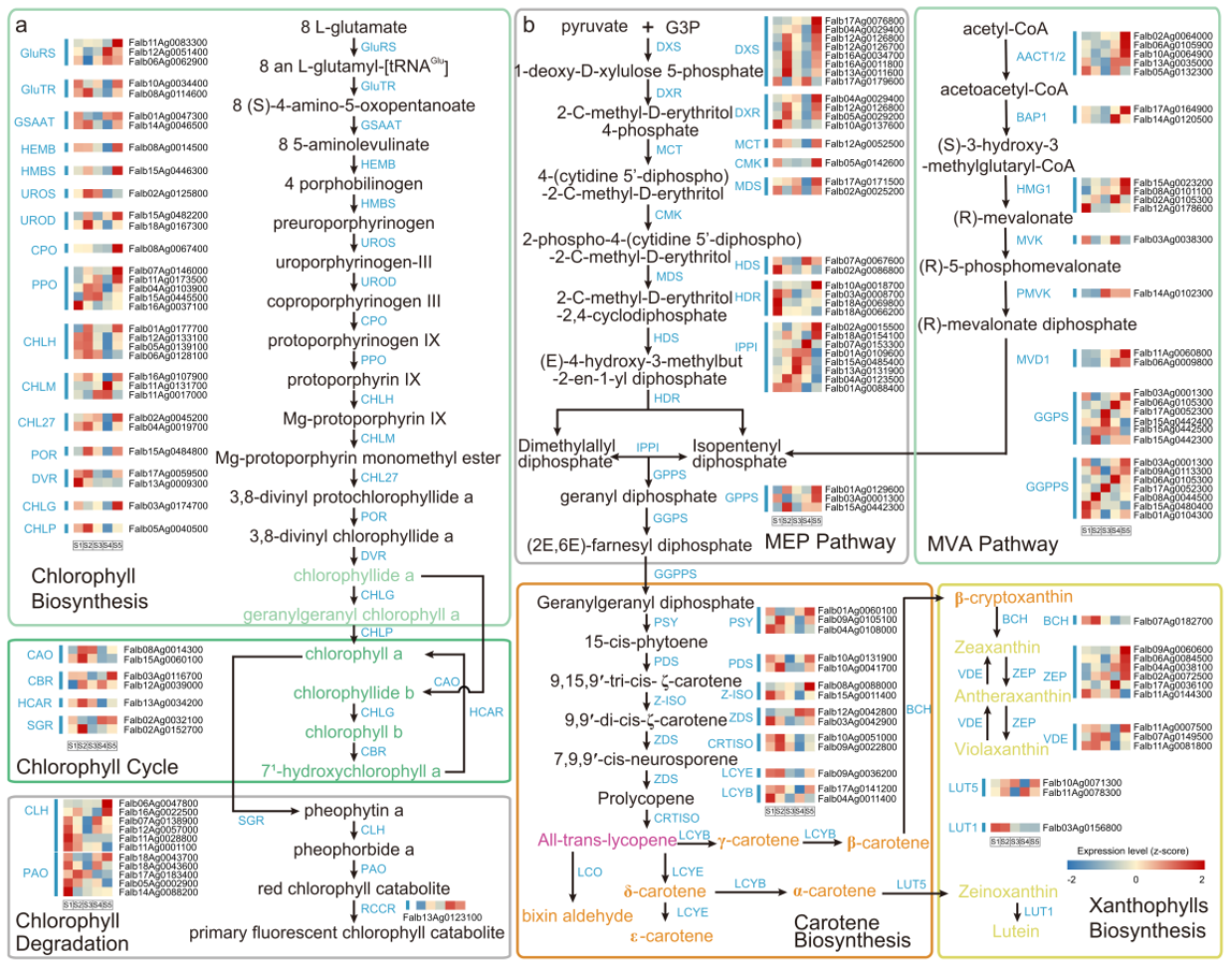
Supplementary Fig.10 Metabolic pathways of carotenoid and chlorophyll biosynthesis, degradation, and cycling in *F. albivenis*. The heatmaps display the gene expression levels (normalized TPMs) across five samples (S1–S5).


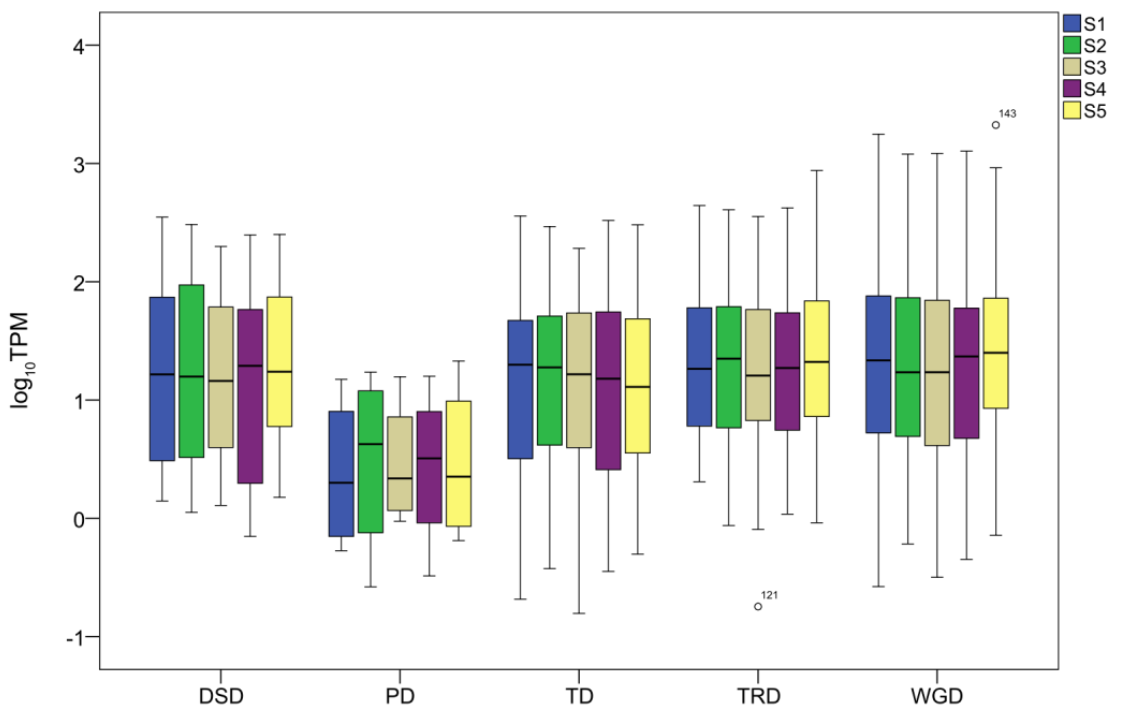


Supplementary Fig. 11 Gene expression levels (log_10_ TPM) of genes derived from different types of duplication events (DSD, PD, TD, TRD, WGD) across five samples (S1–S5). Box plots represent the distribution of expression values within each duplication category.


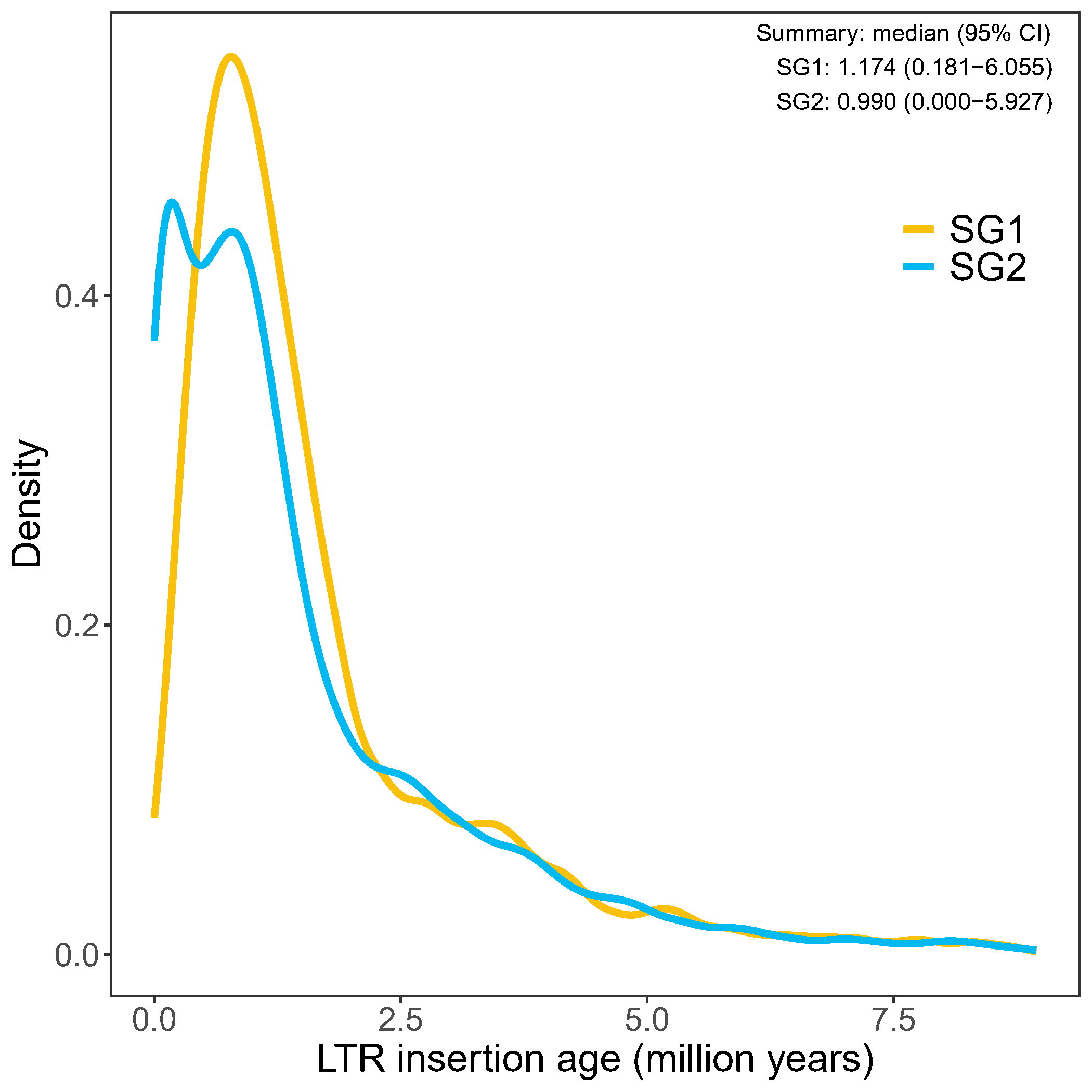
Supplementary Fig. 12 Distribution of LTR retrotransposon insertion ages in subgenomes SG1 and SG2. Density curves show the frequency of LTR insertion events over time.


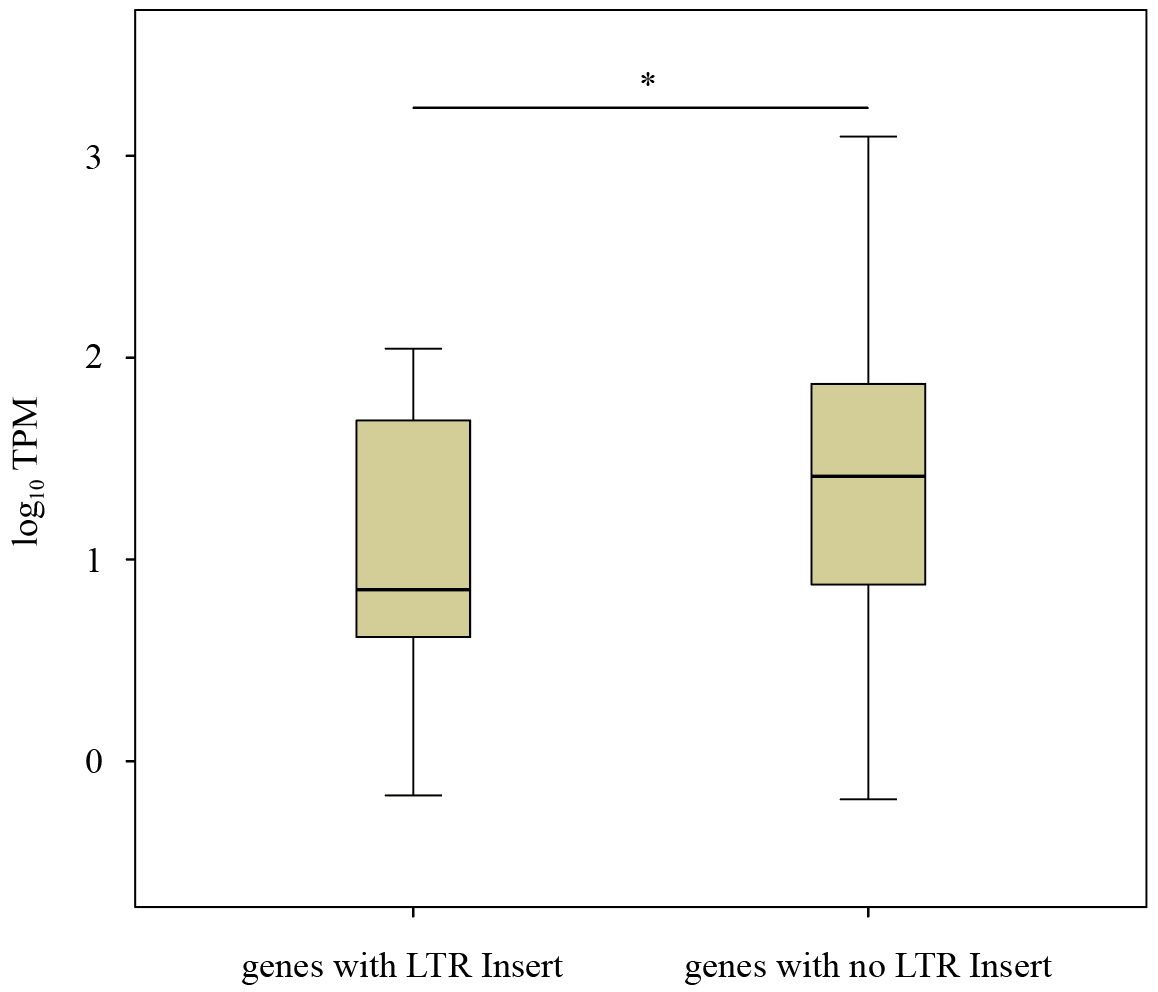


Supplementary Fig. 13 Comparison of gene expression levels (log_10_ TPM) between genes with LTR insertions and genes without LTR insertions. Genes without LTR insertions show higher expression levels (Wilcoxon rank-sum test, *P* < 0.1).


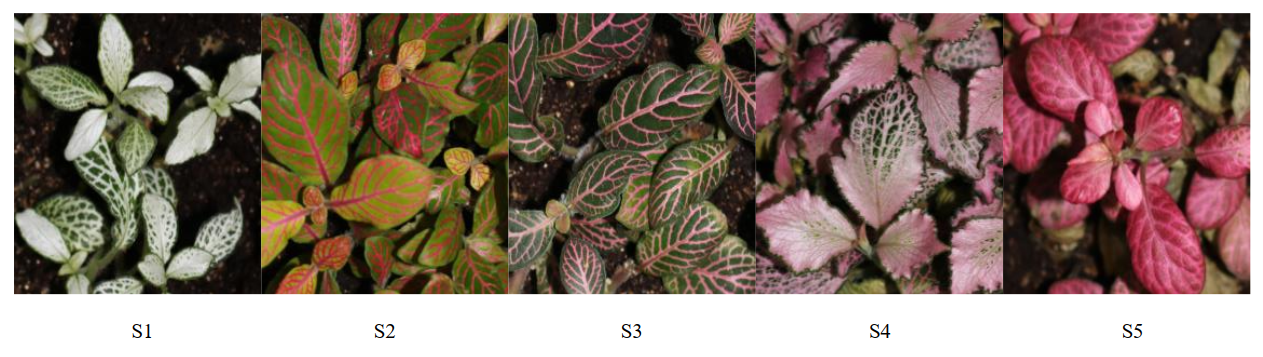
Supplementary Fig. 14 Photographs of five *Fittonia albivenis* accessions (S1–S5) with distinct leaf coloration patterns. These samples correspond to resequencing samples S4, S24, S23, S15, and S17, respectively, as depicted in Fig. 6A.


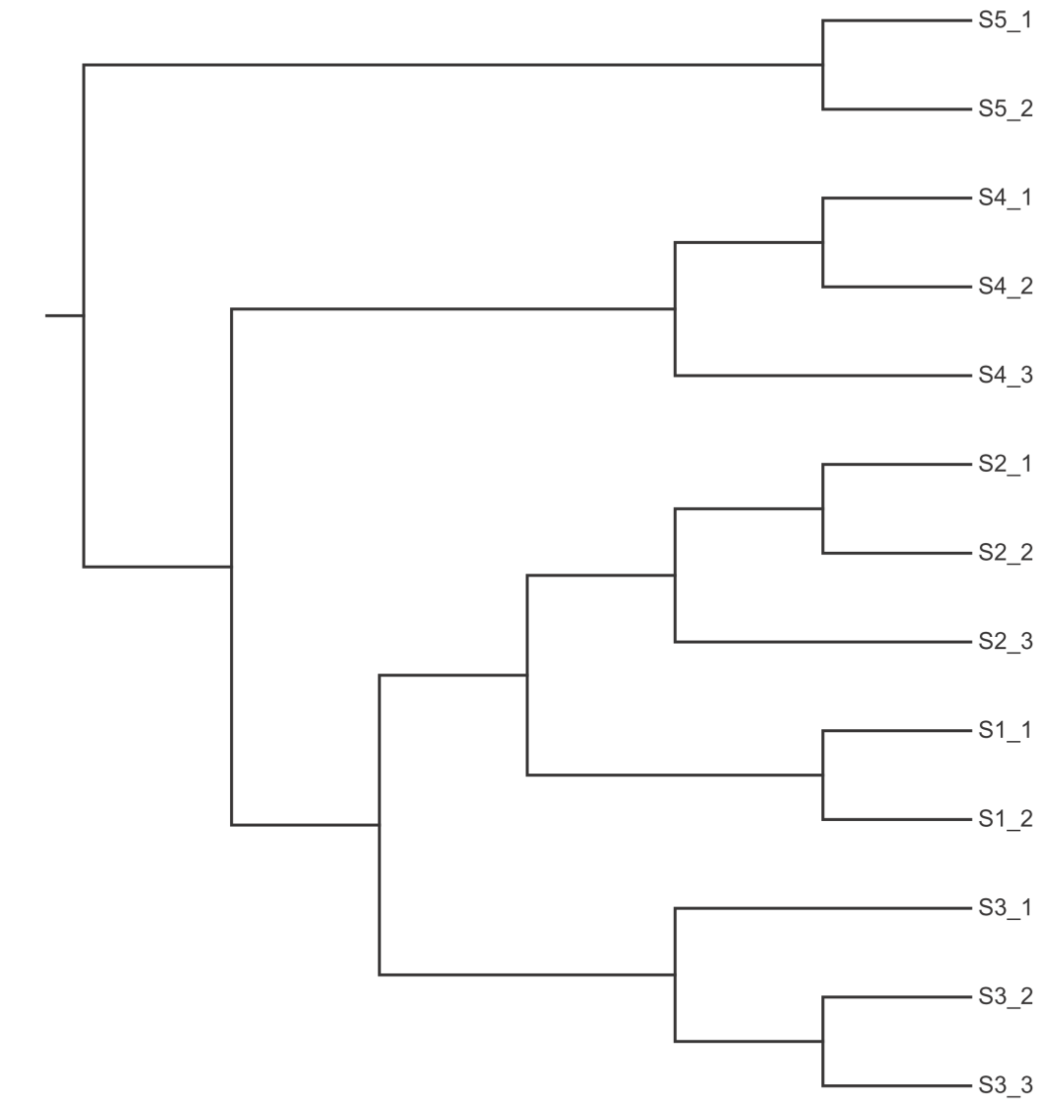
Supplementary Fig. 15 Hierarchical clustering of transcriptomic profiles from five *Fittonia albivenis* accessions (S1–S5), based on normalized gene expression levels (TPM).


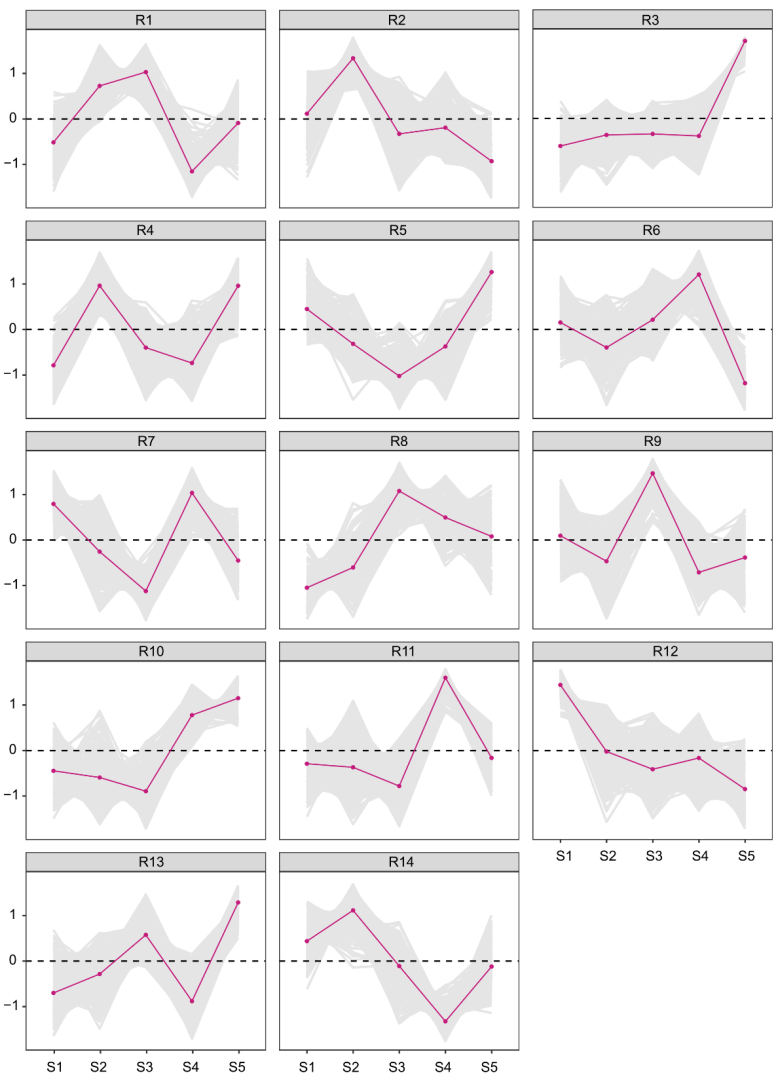
Supplementary Fig. 16 *K*-means clustering of gene expression profiles in *Fittonia albivenis*, resulting in 14 distinct clusters (R1–R14). The X-axis represents five samples (S1–S5), and the Y-axis shows the standardized Z-scores for each gene within each cluster.


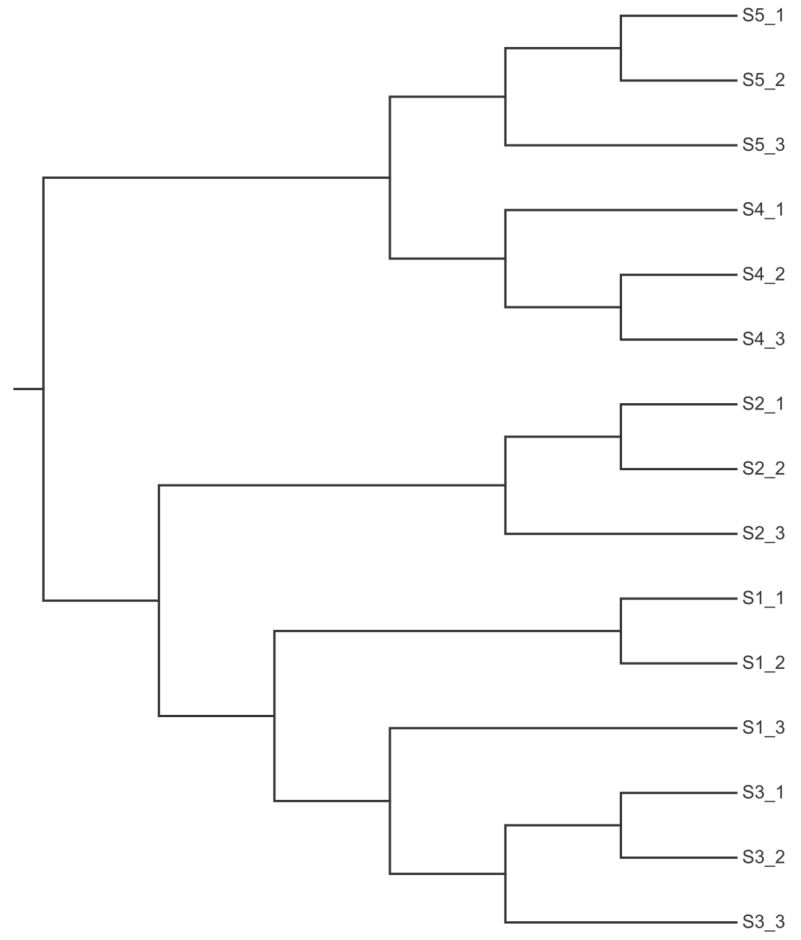
Supplementary Fig. 17 Hierarchical clustering dendrogram of metabolomic profiles across five *Fittonia albivenis* accessions (S1–S5), each with three biological replicates.


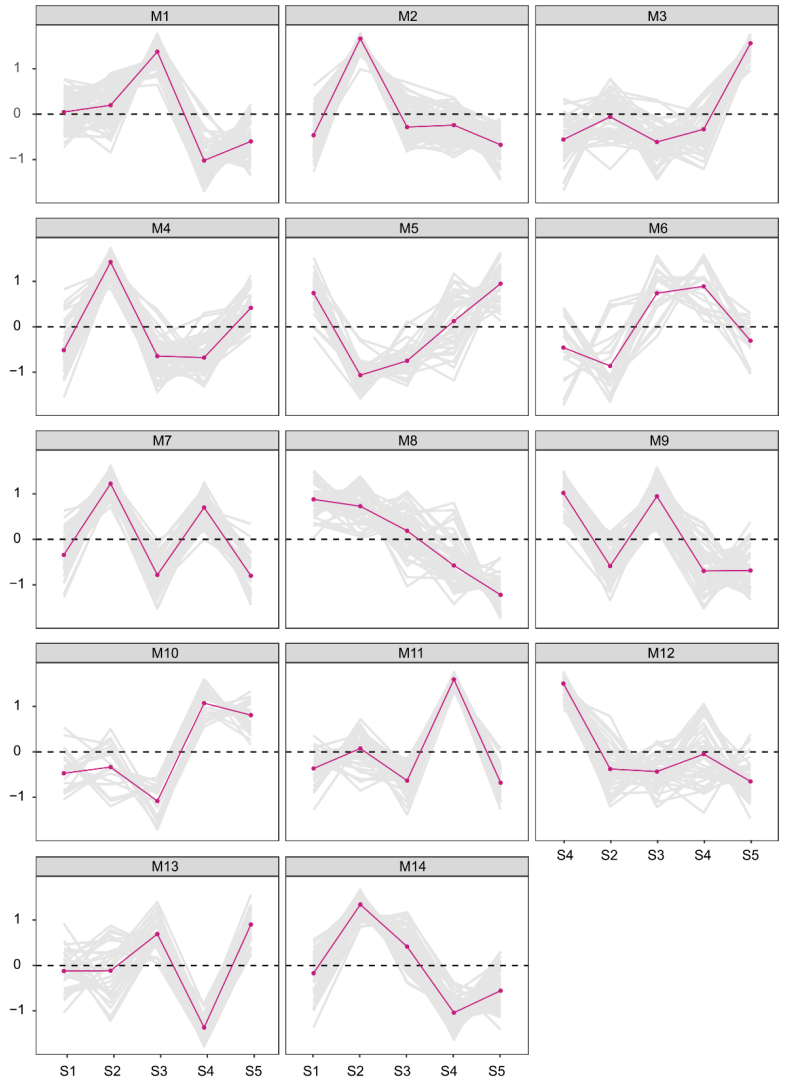
Supplementary Fig. 18 *K*-means clustering of metabolomic profiles in *Fittonia albivenis*, dividing metabolites into 14 distinct clusters (M1–M14) based on standardized Z-scores, revealing dynamic changes in metabolite abundance across five samples (S1–S5).


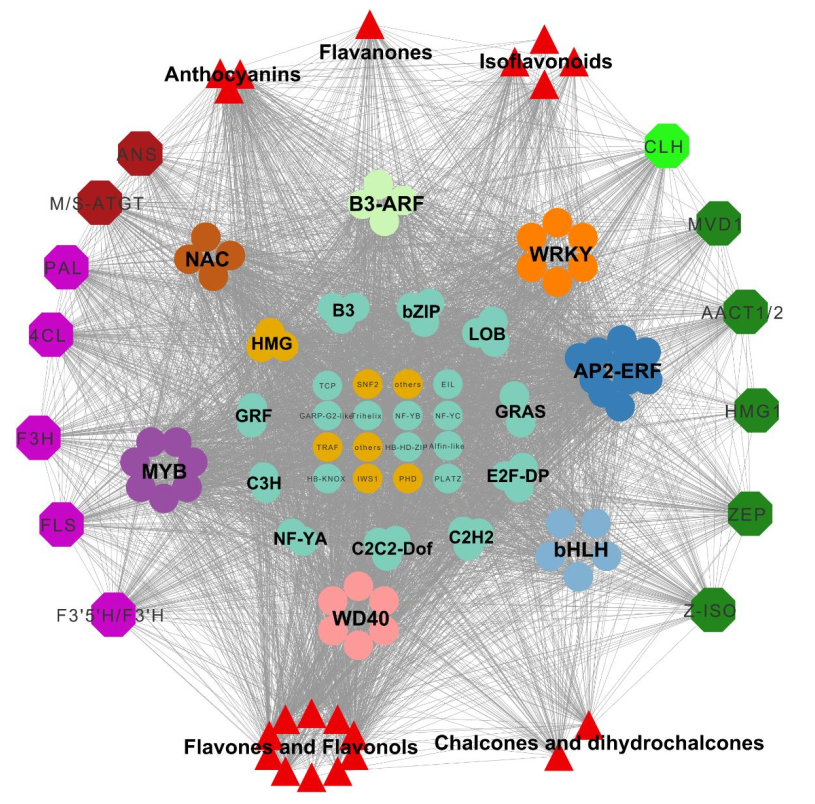


Supplementary Fig. 19 Gene regulatory network constructed based on coexpression analysis. Hexagons colored magenta, red, green, and dark green represent structural genes involved in flavonol, anthocyanin, chlorophyll degradation, and carotene/xanthophyll biosynthesis, respectively. Red triangles denote metabolites associated with flavonol and anthocyanin biosynthesis. Colored circles represent transcription factors from different families regulating these metabolic pathways.


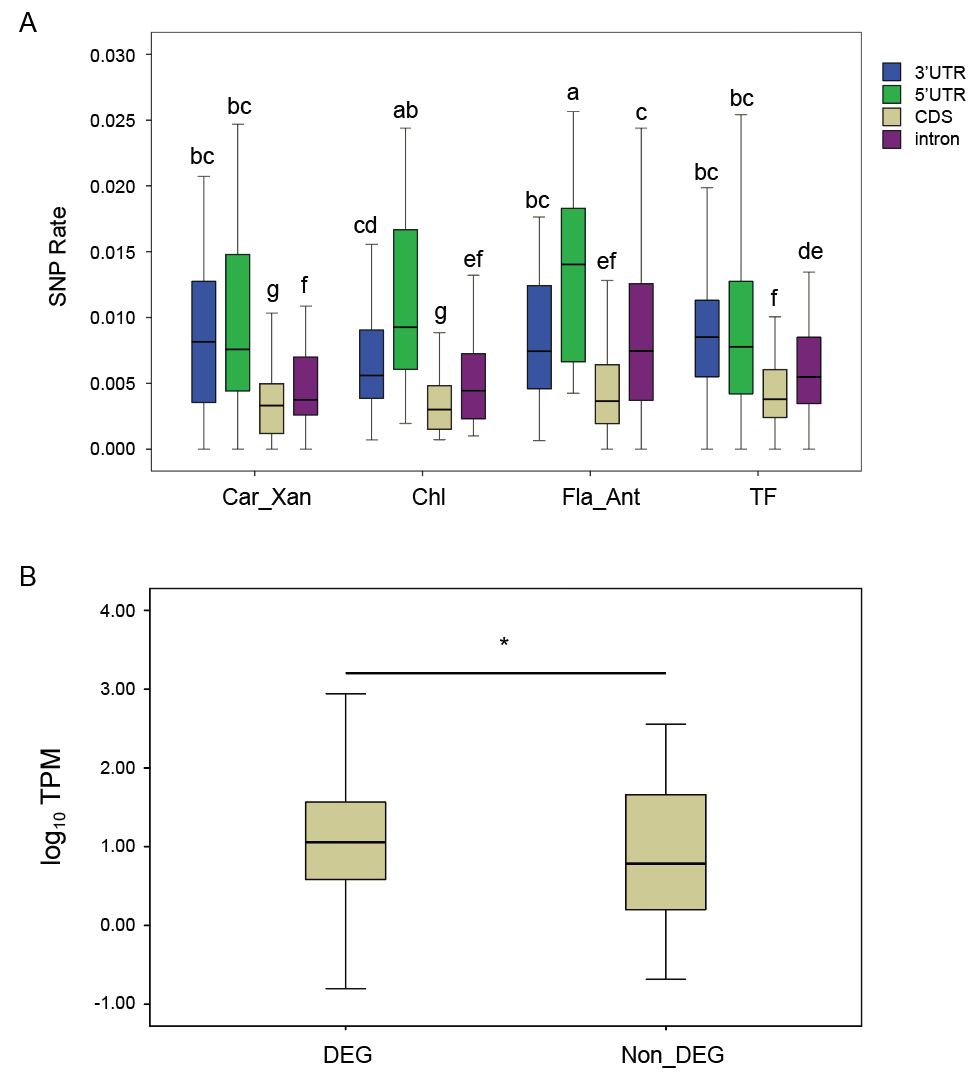
Supplementary Fig. 20 (A) SNP rates across different genomic regions (3’UTR, 5’UTR, CDS, and intron) for genes involved in carotene/xanthophyll biosynthesis (Car_Xan), chlorophyll degradation (Chl), flavonol/anthocyanin biosynthesis (Fla_Ant), and transcription factors (TF). Different letters indicate significant differences between groups (*P* < 0.05, Wilcoxon rank-sum test). (B) Comparison of expression levels (log_10_ TPM) between differentially expressed genes (DEGs) and non-differentially expressed genes (Non_DEGs). * indicates a significant difference (*P* < 0.05, Wilcoxon rank-sum test).
